# RNAs competing for microRNAs mutually influence their fluctuations in a highly non-linear microRNA-dependent manner in single cells

**DOI:** 10.1186/s13059-017-1162-x

**Published:** 2017-02-20

**Authors:** Carla Bosia, Francesco Sgrò, Laura Conti, Carlo Baldassi, Davide Brusa, Federica Cavallo, Ferdinando Di Cunto, Emilia Turco, Andrea Pagnani, Riccardo Zecchina

**Affiliations:** 1 Human Genetics Foundation, Torino, Via Nizza 52, Torino, 10126 Italy; 2 Molecular Biotechnology Center, Università di Torino, Via Nizza 52, Torino, 10126 Italy; 3 Department of Applied Science and Technology, Politecnico di Torino, Corso Duca degli Abruzzi 24, Torino, 10129 Italy; 4 Bocconi University, Milano, Italy; 5 Istituto Nazionale di Fisica Nucleare (INFN) Sezione di, Torino, Italy

**Keywords:** Post-transcriptional cross-regulation, MicroRNA target synchronization, Bimodality, Single cell, Stochastic modelling

## Abstract

**Background:**

Distinct RNA species may compete for binding to microRNAs (miRNAs). This competition creates an indirect interaction between miRNA targets, which behave as miRNA sponges and eventually influence each other’s expression levels. Theoretical predictions suggest that not only the mean expression levels of targets but also the fluctuations around the means are coupled through miRNAs. This may result in striking effects on a broad range of cellular processes, such as cell differentiation and proliferation. Although several studies have reported the functional relevance of this mechanism of interaction, detailed experiments are lacking that study this phenomenon in controlled conditions by mimicking a physiological range.

**Results:**

We used an experimental design based on two bidirectional plasmids and flow cytometry measurements of cotransfected mammalian cells. We validated a stochastic gene interaction model that describes how mRNAs can influence each other’s fluctuations in a miRNA-dependent manner in single cells. We show that miRNA–target correlations eventually lead to either bimodal cell population distributions with high and low target expression states, or correlated fluctuations across targets when the pool of unbound targets and miRNAs are in near-equimolar concentration. We found that there is an optimal range of conditions for the onset of cross-regulation, which is compatible with 10–1000 copies of targets per cell.

**Conclusions:**

Our results are summarized in a phase diagram for miRNA-mediated cross-regulation that links experimentally measured quantities and effective model parameters. This phase diagram can be applied to in vivo studies of RNAs that are in competition for miRNA binding.

**Electronic supplementary material:**

The online version of this article (doi:10.1186/s13059-017-1162-x) contains supplementary material, which is available to authorized users.

## Background

MicroRNAs (miRNAs) are small non-coding post-transcriptional repressors of gene expression [1]. They exert important regulatory functions on both protein-coding and non-coding genes and are often involved in pivotal biological processes, like developmental biology or the molecular pathogenesis of several diseases [2–5]. It is commonly believed that miRNAs play central roles in conferring robustness to biological processes against environmental fluctuations [6–10]. The common assumption that at any given time one miRNA molecule can interact at most with one target mRNA [11] suggests a whole new layer of post-transcriptional cross-regulation, lately named the *competing endogenous RNA (ceRNA) effect* [12]. This theory proposes that the amount of a gene product may be tuned by varying the concentration of another transcript that shares with it the same miRNAs. Qualitative experiments based on observing induced variations in the level of transcripts show indeed that these could be coupled, due to the interaction with a common pool of miRNAs [13–16]. The discovery that the miRNA–target interaction is compatible with a titration mechanism [17] supports the emergence of hypersensitivity regions [18, 19] where miRNA targets should be highly correlated and their relative stoichiometry tightly controlled [20–22]. Many biochemical competition phenomena that are qualitatively like the one studied here have been extensively studied in the past [23–25]. However, the relevance of competition at the post-transcriptional level is still largely debated [26]. Indeed, on the one hand, absolute quantification experiments in primary hepatocytes and liver suggested that the ceRNA effect is unlikely to affect significantly gene expression and metabolism [27]. On the other hand, differential susceptibility based on endogenous miRNA/target pool ratios provide a physiological context for target competition in vivo [28]. Moreover, recent studies on topics ranging from development [29] to cancer [30–34] show how the competition for miRNA binding can actively regulate key biological processes. Crosstalk among mRNAs may, thus, be regulated depending on miRNA and mRNA relative abundances and may exhibit non-negligible target correlation profiles [20, 21].

Here, we experimentally explore these features and address the relevance of the relative mRNA–miRNA stoichiometric composition. In particular, we set out to validate a stochastic gene interaction model [20] that describes how mRNAs sharing common miRNA regulatory elements (MREs) can influence each other’s fluctuations in a miRNA-dependent way. Thus, we designed two bidirectional plasmids, each with a two-color fluorescent reporter system, enabling the simultaneous tracking of gene expression in the presence and absence of MREs. This allows us to quantify the correlations between the expression of the encoded proteins under different conditions. We found that there is an optimal range of parameters (in terms of effective transcription rates and miRNA interaction strengths) for which cross-regulation is maximal among miRNA targets. We show that such cross-regulation arises both at the level of mean protein concentrations (like [35]) and, for the first time to the best of our knowledge, at the level of fluctuations and correlations.

We show that the optimal cross-regulation regime is compatible with low numbers of mRNA molecules. In particular, the bulk quantification of mean exogenous transcripts per cell reveals that in our experiments, the crosstalk is highest in a physiological regime of order 10 to 1000 molecules per cell [36, 37]. Interestingly, in agreement with the model, we found that the same mechanism may induce bimodal population distributions with distinct high and low expression states of the targets.

## Results

### Stochastic titration model for crosstalk

**Fig. 1:**
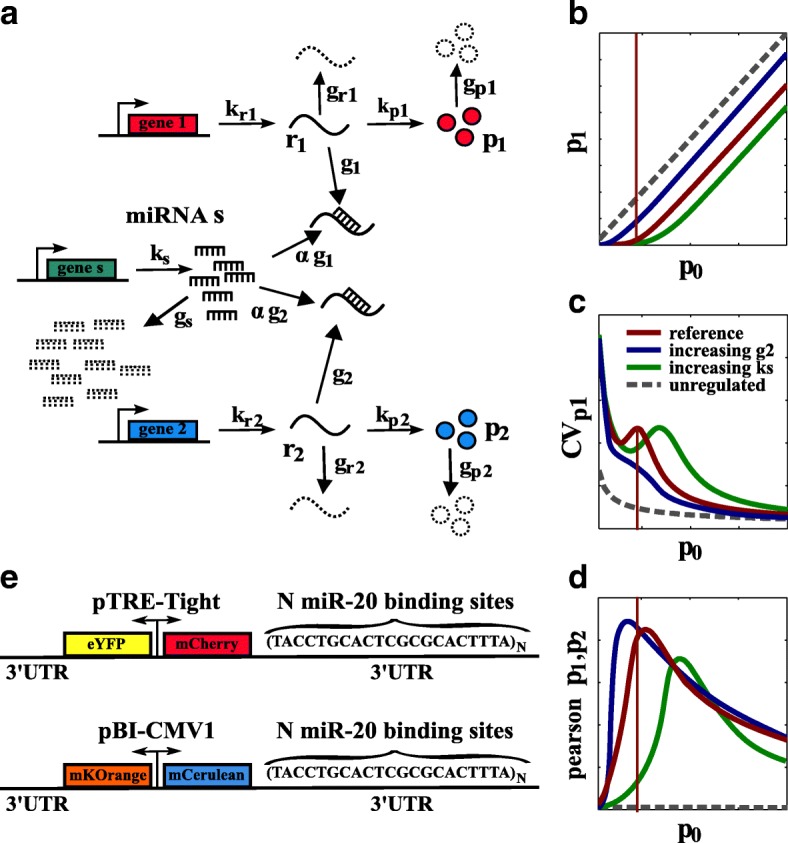
Model and predictions. **a** Sketch of the minimal model of miRNA–target interactions. One miRNA *s* and two targets *r*_1_ and *r*_2_ are independently transcribed with rates *k*_*s*_, $k_{r_{1}}$, and $k_{r_{2}}$, respectively. Each transcript can then degrade with rate *g*_*s*_, $g_{r_{1}}$, or $g_{r_{2}}$, respectively. Each miRNA *s* can interact with targets *r*_1_ or *r*_2_ with effective binding rates *g*_1_ or *g*_2_. *α* measures the probability of miRNA recycling. If not bound to a miRNA, targets *r*_1_ and *r*_2_ can be translated into proteins *p*_1_ and *p*_2_, respectively, which could then degrade with rates $g_{p_{1}}$ and $g_{p_{2}}$. **b**–**d** Predictions from the stochastic model of interactions sketched in (**a**) as a function of *p*_0_ (which is the constitutive value of *p*_1_ when *g*_1_ tends to 0) in terms of **b** the mean amount of *p*_1_ free molecules, **c** the *p*_1_ coefficient of variation ${CV}_{p_{1}}$, and **d** the Pearson correlation coefficient between *p*_1_ and *p*_2_. In (**b**–**d**), the *red curve* is the reference curve for a given set of parameters while the *red line* identifies the threshold. *Blue* and *green curves* show how the *red curve* would move when increasing the interaction strength with the second target *g*_2_ or the pool of miRNA via the miRNA transcription rate *k*_*s*_, respectively. **e** Schematic representation of the two bidirectional plasmids coding for the four fluorophores. *miRNA* microRNA, *UTR* untranslated region

We propose a stochastic model for the miRNA-mediated target cross-regulation [17, 20, 23] (see Fig. 1 a). Through the formulation of a chemical master equation (see “Methods” and Additional file 1 for details of the model), the model describes the mean amount and fluctuations of two mRNAs *r*_1_ and *r*_2_, which are both targets of the same miRNA *s*. Both *r*_1_ and *r*_2_ can be translated into proteins (*p*_1_ and *p*_2_, respectively) only when not bound to the miRNA. The mRNA–miRNA complex can be degraded as a whole, while the miRNA can be recycled with probability 1−*α*. Since the two targets *r*_1_ and *r*_2_, and thus *p*_1_ and *p*_2_, are coupled through their common regulatory miRNA *s*, the pool of available mature miRNAs is the limiting factor in a system of potentially interacting targets.

A Gaussian approximation of the master equation [20] (see Additional file 1) allows us to evaluate mean values (〈*x*〉), noise (coefficient of variation *CV*_*x*_=*σ*_*x*_/〈*x*〉), and Pearson correlation coefficients (*ρ*_*x,y*_=(〈*x y*〉−〈*x*〉〈*y*〉)/*σ*_*x*_ *σ*_*y*_) for each molecular species *x* represented in Fig. 1 a (with *x*∈{*r*_1_, *r*_2_, *p*_1_, *p*_2_, *s*}); see Fig. 1 b–d, respectively. The parameters *g*_1_ and *g*_2_, which are proportional to the miRNA–mRNA association rate, determine qualitatively the shapes of the functions generated by the model. In Fig. 1 b–d, these curves are plotted as a function of protein constitutive expression *p*_0_ (i.e., the value of *p*_1_ or *p*_2_ when *g*_1_ or *g*_2_ tends to 0). When one of these parameters tends to zero (say *g*_2_), its corresponding target (*r*_2_) is not interacting with the miRNA. The other target *r*_1_ (and, thus, *p*_1_) is repressed until a threshold level of *r*_0_ (and, thus, *p*_0_) is exceeded (Fig. 1 b) [17]. The threshold is established by miRNA regulation and its location can be adjusted by regulating both the pool of miRNAs (via the miRNA transcription rate *k*_*s*_) and the pool of targets (via the target transcription rates $k_{r_{1}}$ and $k_{r_{2}}$) [17, 20]. The increase of *g*_1_ sharpens the transition between threshold and escape regimes. From the point of view of *r*_1_, *g*_2_ (proportional to the association constant of the second target) governs the concentration of free miRNA available within the cell. Increasing *g*_2_ (while keeping all other parameters fixed) pushes the threshold to lower values of expression (lower *r*_0_ and *p*_0_) and globally increases *r*_1_ (and *p*_1_). *r*_2_ behaves as a sponge for the miRNA, and increasing *g*_2_ is equivalent to sponging away the miRNA available to target *r*_1_. When all the miRNAs have been sponged away by *r*_2_ (high value of *g*_2_), then *r*_1_ is not regulated anymore. At intermediate conditions in which miRNA is not completely sponged away by one of the targets, finely tuned cross-regulation between targets is possible. The mathematical model, thus, suggests experiments for testing this hypothesis and quantifying the crosstalk, modulated by *g*_1_, *g*_2_, and the amount of miRNA present in the cell.

### Experimental set-up for unraveling cross-regulation

To investigate the predicted miRNA-mediated cross-regulation in single mammalian cells, we transfected two different two-color fluorescent reporters (sketched in Fig. 1 e) in the HEK 293 cell line. Both constructs consist of bidirectional promoters driving two genes whose products are fluorescent proteins. The first construct expresses the fluorescent proteins mCherry and enhanced yellow fluorescent protein (eYFP) [17], while the second construct expresses mCerulean and mKOrange. The 3^′^ untranslated region (UTR) of both mCherry and mCerulean was engineered to contain a fixed number *N* of MREs for miR-20a (with *N*=0,1,4,7), a miRNA endogenously expressed by the HEK 293 cell line [38, 39] and related to cell proliferation.

mCherry and mCerulean are, therefore, proxies for the two targets in the model (*p*_1_ and *p*_2_). The 3^′^ UTRs of eYFP and mKOrange were left unchanged in order to measure the transcriptional activity of the reporters in single cells (they are proxies for *p*_0_). The constructs, thus, allow simultaneous monitoring of protein levels with (mCherry and mCerulean) and without (eYFP and mKOrange) miRNA regulation. The absence of a control on the number of plasmids per cell allows us to explore the variation of target transcription levels by simply sorting the cells on their eYFP or mKOrange fluorescence level.

For single-construct transfections, we observed a threshold effect [17]. Briefly, when no MREs are present, the levels of expression of mCherry (mCerulean) and eYFP (mKOrange) are proportional. In cells with one or more miR-20a site on mCherry (mCerulean), the mCherry (mCerulean) level does not increase until a threshold level of eYFP (mKOrange) is exceeded (see Additional file 1: Figure S1). This indicates that protein production is highly repressed below the threshold established by miRNA regulation and responds sensitively to target mRNA input close to it. Cotransfections of both constructs with different MRE numbers and measurements of fluorescence with a flow cytometer enabled the quantification of the crosstalk between mCherry and mCerulean as a function of *N*. Different combinations of MRE numbers mimic the variation of the model parameters *g*_1_ and *g*_2_. To capture the cross-regulation quantitatively, we measured the joint distributions of mCherry (*p*_1_) and eYFP (*p*_0_) levels given mCerulean (*p*_2_) in single cells positive to the fluorophores. We, therefore, binned the data according to their eYFP levels and calculated the mCherry and mCerulean mean levels as well as their standard deviations and Pearson correlation coefficients in each eYFP bin. To show that our results are unbiased with respect to the cell line we used, we repeated the experiments in HeLa cells. These data are presented in Additional file 1.

### The extent of cross-regulation is determined by relative miRNA/target stoichiometry

**Fig. 2:**
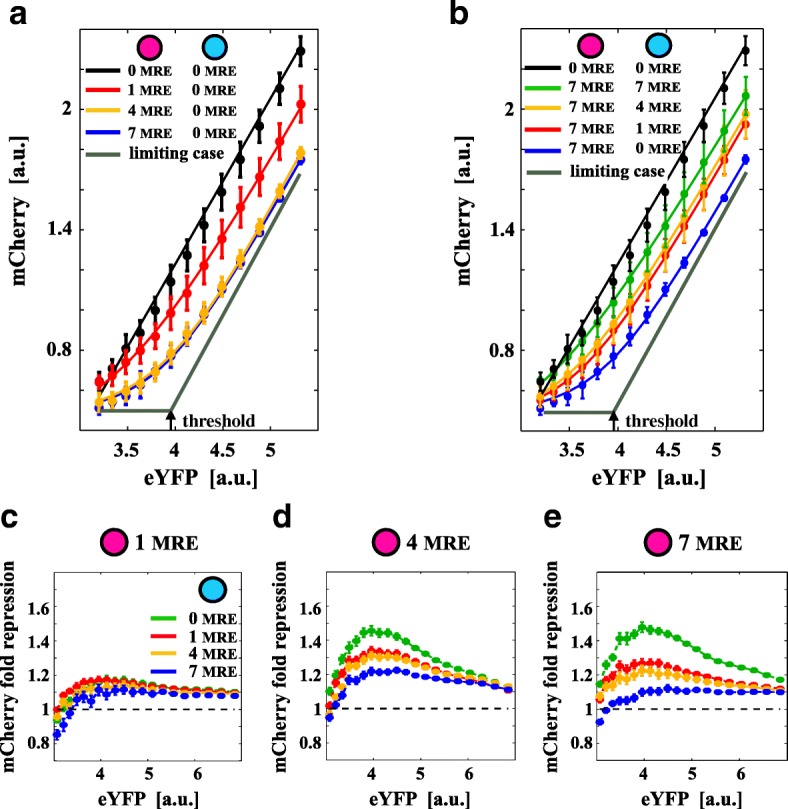
Titration-induced threshold determines the optimal crosstalk. **a**, **b** mCherry mean fluorescence (a proxy for *p*_1_ in the model, Fig. 1 b) is plotted against eYFP (a proxy for the constitutive expression *p*_0_ in the model). *Error bars* are evaluated on the biological replicates. *Continuous lines* are model fits. The *gray curves* in (**a**) and (**b**) are the model prediction with the parameters fitted from the data and miRNA/target effective interaction strength *g*_1_→*∞*. The *black arrow* points to the model-predicted threshold. A threshold (or non-linear behavior) emerges when increasing mCherry MRE (**a**) while it disappears when increasing mCerulean MRE (**b**). The onset of the threshold is very close to the origin of the plot, indicating a relatively small amount of free miRNA. The intensity of crosstalk (measured in terms of fold-repression *F* with respect to the unregulated fluorophores) depends on the particular combination of MRE on both exogenous targets (**c**–**e**). *F* is the ratio between the value of mCherry in the absence of miR-20a MREs and its value in the presence of MREs for each eYFP bin and for each *N* on mCerulean. *Purple* and *cyan circles* in legends represent the plasmids coding for the mCherry and mCerulean fluorophores. *a.u.* arbitrary units, *eYFP* enhanced yellow fluorescent protein, *MRE* miRNA regulatory element

To quantify the crosstalk by modulating *g*_1_ and *g*_2_, we expressed different numbers of MREs on mCherry and mCerulean (*N*=1,4,7) and compared them when *N*=0. These cotransfections allowed us to follow the expression of one target (mCherry) while tuning the amount of free miRNA via the second target (mCerulean). As predicted by the model (Fig. 1 b), it is possible to identify two different effects: (i) the appearance of a threshold on mCherry while increasing the number *N* of MREs on its 3^′^ UTR and keeping *N*=0 on mCerulean (Fig. 2 a and Additional file 1: Figure S2a) and (ii) a global increase of the mCherry mean fluorescence and a shift in the threshold while increasing *N* on mCerulean (Fig. 2 b and Additional file 1: Figure S2b). Threshold effects such those shown in Fig. 2 are a typical landmark for non-linear behavior. More specifically, the strength of the miRNA–target interaction dictates the departure from linear behavior. In the absence of an miRNA–target interaction, the mean number of transcripts (and proteins) miRNA-mediated regulation breaks this linear dependence by inducing highly non-linear threshold effects.

In Fig. 2 a, b, circles are data points with error bars over the experimental replicates while continuous lines are model fits (see Additional file 1 for details). The gray curves in Fig. 2 a and b are the model prediction with the parameters fitted from the data and *g*_1_→*∞*. Notice that the onset of the threshold is very close to the origin of the plot, indicating a relatively small amount of free miRNA. mCherry tends to the unregulated case (mCherry is linearly proportional to eYFP) on increasing the number of MREs on mCerulean. This result is well summarized by the fold-repression *F* between regulated and unregulated mCherry mean fluorescence (Fig. 2 c–e). *F* is the ratio between the value of mCherry in the absence of miR-20a MREs and its value in the presence of MREs for each eYFP bin and for each *N* on mCerulean. Increasing the number of MREs on mCherry increases its repression, and *F* is highest when mCerulean has *N*=0 MREs while it tends to one on increasing the eYFP expression or the number of MREs on mCerulean. In particular, near the threshold, *F* shows a maximum whose value depends both on mCherry and mCerulean MREs. *F* could be indirectly considered as a measure of cross-regulation between the two targets.

These data show that the cross-regulation is maximal near the threshold and for intermediate levels of repression (in our case when mCerulean is between one and four MREs).

### miRNA increase shifts the optimal cross-regulation region

**Fig. 3:**
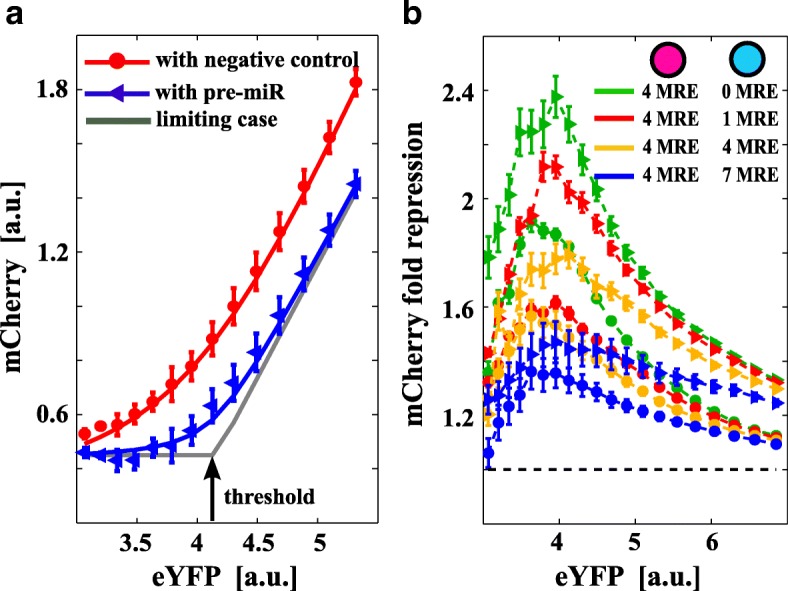
miRNA increase shifts the maximal crosstalk region. **a** mCherry mean fluorescence (a proxy for *p*_1_ in the model, Fig. 1 b) is plotted against eYFP (a proxy for the constitutive expression *p*_0_ in the model). *Blue triangles* and *red circles* are data from cotransfection with pre-miR20a and negative controls, respectively. *Error bars* are evaluated on the biological replicates. The *gray curve* is the model prediction with the parameters fitted from the data and miRNA/target effective interaction strength *g*_1_→*∞*. The *black arrow* points to the model-predicted threshold. According to the model, increasing the pool of available miRNAs (transfecting pre-miRNAs) shifts the threshold to higher constitutive expression values. **b** Different combinations of miR-20a MREs lead to different levels of fold-repression and crosstalk. *Triangles* and *circles* in the plot are data from transfections with pre-miR20a and negative controls, respectively. *Purple* and *cyan circles* in legends represent the plasmids coding for mCherry and mCerulean fluorophores, respectively. *a.u.* arbitrary units, *eYFP* enhanced yellow fluorescent protein, *MRE* miRNA regulatory element

To assess the cross-regulation dependence on the availability of miRNA, we transfected 100 nM of pre-miR for miR-20a together with the bidirectional constructs. In our model, this is equivalent to increasing the basal miRNA transcription rate *k*_*s*_. We analyzed the cases with *N*=4 for mCherry and *N*=0,1,4,7 for mCerulean. In agreement with the model predictions (see Fig. 1 b), we observed a shift of the threshold towards higher eYFP levels (Fig. 3 a) together with a global increase in the fold-repression (Fig. 3 b) and a resulting shift of the optimal crosstalk region towards a higher number of MREs. In Fig. 3 a, triangles and circles are data points for cotransfections with pre-miR20a and negative controls, respectively, while continuous lines are model fits (see Additional file 1 for details). The gray curve in Fig. 3 a is again the model prediction with the parameters fitted from the data and *g*_1_→*∞*.

We then quantified by quantitative PCR the mean absolute number of exogenous targets in three subpopulations of cells (bulk measurements), sorted according to their eYFP intensity (low, medium, or high) both in the presence and absence of pre-miR for when *N*=4 on mCherry and *N*=1 on mCerulean (Table 1). We found that mCherry and mCerulean ranged from 40 to 400 and from 10 to 200 mean molecules per cell, respectively, without pre-miR and both from 10 to about 250 mean molecules in the presence of pre-miR (see Additional file 1 for details). Mature miR-20a, both endogenous and in cells transfected with 100 nM of its pre-miR, was quantified as well. We found about 1250 molecules per cell of mature miR-20a in the untransfected cells and 163 times more mature molecules in the pre-miR transfected cells (see Additional file 1 for details).

**Table 1:** Absolute quantification of exogenous targets

Exogenous transcript	Cotransfection	Cotransfection + pre-miR-20a
eYFP low	58 (42)	41 (28)
eYFP medium	354 (398)	103 (54)
eYFP high	2986 (2840)	336 (28)
mCherry low	34 (18)	10 (9)
mCherry medium	113 (50)	21 (18)
mCherry high	424 (368)	205 (214)
mCerulean low	6 (1)	8 (3)
mCerulean medium	60 (54)	36 (29)
mCerulean high	208 (152)	261 (296)

Absolute quantification of the number of transcripts per cell for the exogenous molecules (standard deviation over the biological replicates in parentheses) for the cotransfection of the two constructs (second column), and the cotransfection of the two constructs plus pre-miR20 (third column). The mean number of mRNA exogenous molecules for three different intervals of eYFP basal expression is low enough to be comparable with physiological values

These data show that in our system, the maximal cross-regulation region is dependent on the relative number of both miRNAs and targets and it is compatible with a low number of mRNA molecules.

### Titration induces increased cell-to-cell variability and bimodality

It is well known that the intrinsic noise of an unregulated gene product decreases when its expression level increases [40]. The effect of miRNA regulation introduces an extra source of noise (extrinsic noise). Our mathematical model predicts that, at fixed levels of expression, the total noise (intrinsic plus extrinsic) of a miRNA-regulated gene product (say ${CV}_{p_{1}}$) should increase or decrease upon enhancing miRNA–target interaction strengths *g*_1_ or *g*_2_, respectively (see Fig. 1 c), compared to the unregulated case (when *g*_1_→0). The increase of ${CV}_{p_{1}}$ is due to the coupling of the intrinsic noise of the target *r*_1_ (and, thus, *p*_1_) to the extrinsic noise of the miRNA *s* induced by the titration reactions. Increasing the interaction strength of the second target *g*_2_ partially decouples *r*_1_ and *s*, thus, reducing the noise ${CV}_{p_{1}}$. In particular, the model predicts the onset of a local maximum in the noise profile of the miRNA target versus its level of constitutive expression for high *g*_1_ near the threshold. Indeed, near the threshold, also the coupling between the two targets *r*_1_ and *r*_2_ (and, thus, *p*_1_ and *p*_2_) becomes non-negligible (maximal cross-regulation) and contributes to the total noise. The local maximum is a vivid manifestation of the so-called retroactivity phenomenon, i.e., of how binding and titration can introduce correlations between intrinsic and extrinsic noise [41]. The intrinsic noise of one target is coupled to the extrinsic noise of the miRNA and in turn to the extrinsic noise of the other miRNA target.

**Fig. 4:**
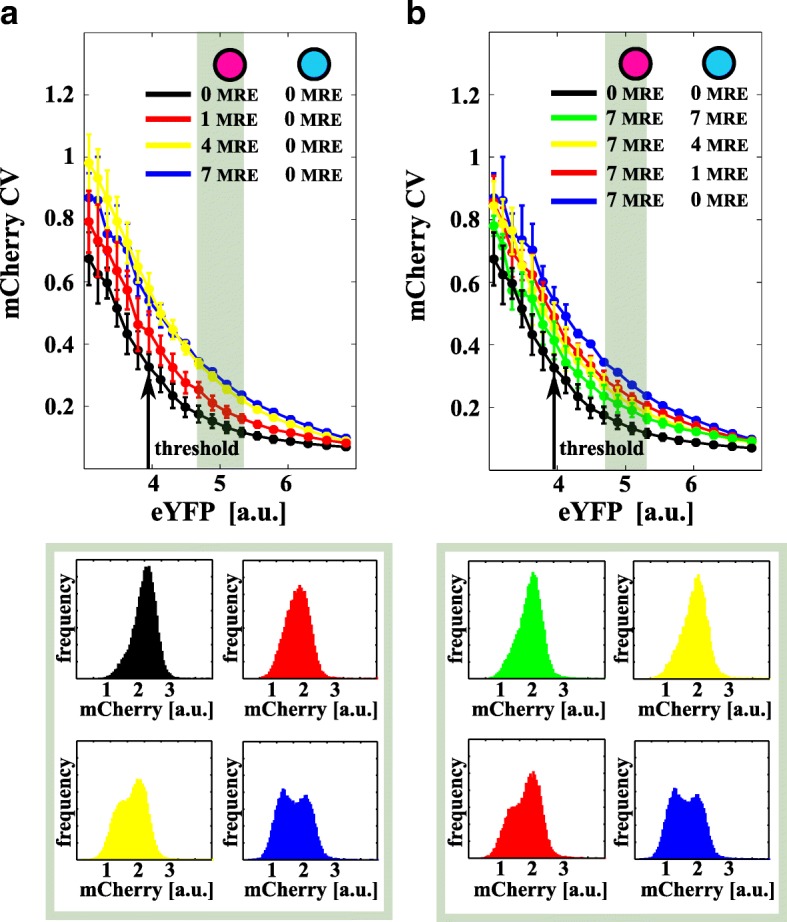
Retroactivity increases cell-to-cell variability. **a**, **b** mCherry total noise, quantified by its coefficient of variation (CV, a proxy for ${CV}_{p_{1}}$ in Fig. 1 c), is plotted against eYFP (a proxy for the constitutive expression *p*_0_ in the model). The *black arrow* identifies the model-predicted threshold shown in Fig. 2. *Error bars* are evaluated on the biological replicates. CV globally increases on increasing the number of mCherry MREs (**a**) while it decreases on increasing the number of mCerulean MREs (**b**). The competition between these two strengths results in lowering the noise even if the expected repression from the rough number of mCherry MREs is high. Histograms in the lower panels show mCherry data distributions for the shaded regions in (**a**) and (**b**). A strong miRNA target repression strength increases cell-to-cell variability with the eventual appearance of different phenotypes (bimodal distributions). *Purple* and *cyan circles* in legends represent the plasmids coding for mCherry and mCerulean fluorophores, respectively. *a.u.* arbitrary units, *CV* coefficient of variation, *eYFP* enhanced yellow fluorescent protein, *MRE* miRNA regulatory element

Experimentally, we show that: (i) upon increasing *N* on mCherry (i.e., *g*_1_), the total noise of mCherry globally increases as a function of eYFP (Fig. 4 a and Additional file 1: Figure S2c) and (ii) upon increasing *N* on mCerulean (i.e., *g*_2_), the total noise of mCherry globally decreases (Fig. 4 b and Additional file 1: Figure S2d). For high levels of repression (high *N* on mCherry and low *N* on mCerulean), the mCherry CV eventually shows a local maximum near the threshold (Additional file 1: Figure S2c, d). A low level of noise indicates unimodal distributions while an increase in noise indicates increased cell-to-cell variability and may indicate bimodal population distributions with distinct high and low expression states [42]. We then checked if this was the case and found that bimodality on mCherry is present near the threshold for a high miRNA–target interaction (*N*=4,7 on mCherry and *N*=0,1 on mCerulean); see the histograms in Fig. 4 and Additional file 1: Figure S3. In particular, for *N*=7 on mCherry and *N*=0 on mCerulean, two very discernible phenotypes appear. This suggests the binary response is directly linked to the variability in the level of repression the miRNA exerts on the target [20]. Near the threshold, where the numbers of free target and miRNA molecules are small and similar, stochastic fluctuations become decisive for the cell fate. A small fluctuation in the number of miRNAs or targets will indeed produce cells with a highly repressed or unrepressed target product depending on the particular miRNA repression strength exerted on the target.

Our data show that miRNA–target titration reactions introduce non-trivial couplings between miRNA and targets (retroactivity) that possibly result in an increase in noise and bimodal cell population distributions near the threshold.

### Titration-induced retroactivity causes target synchronization

**Fig. 5:**
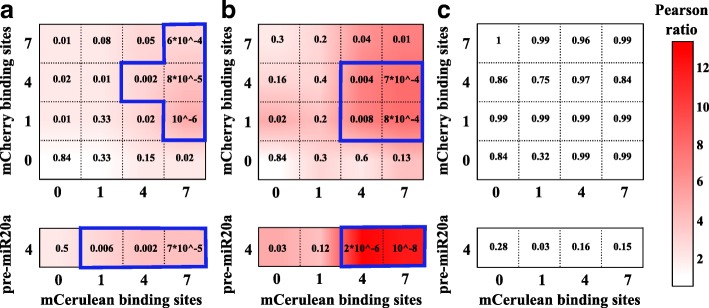
Fold Pearson and *p* values. The Pearson ratio is measured for three different values of eYFP basal expression: below threshold (**a**), around threshold (**b**), and above threshold (**c**). *p* values are reported for each combination of miRNA MREs on the two plasmids. The regions inside the *blue perimeters* are statistically significant with *p*<0.01. As predicted by the model, the correlation is maximal around the threshold and could be even 12-fold higher than in the unregulated case. *Blue-delimited areas* are regions whose Pearson ratio (i.e., the ratio of the Pearson coefficients between mCherry and mCerulean possessing different MREs for the same measure in the absence of MREs) is statistically relevant with respect to the corresponding unregulated case. *eYFP* enhanced yellow fluorescent protein, *miRNA* microRNA, *MRE* miRNA regulatory element

The model predicts a maximum in the correlation between the two target products *p*_1_ (mCherry) and *p*_2_ (mCerulean) near the threshold (see Fig. 1 d). We investigated the strength of this prediction, distinguishing between correlations dependent on the experimental setting (mainly transient cotransfections and partial sharing of regulatory elements in the promoter) and correlations induced by the competition for miRNA binding, which can potentially lead to synchronized fluctuations. We, thus, defined the ratio of the Pearson correlation coefficients (the ratio of the Pearson coefficients between mCherry and mCerulean possessing different MREs for the same measure in the absence of MREs). We measured this ratio for eYFP below, around, and above the threshold (Fig. 5 a–c, respectively), and observed that the competition for miRNA binding introduces correlations ranging from 4- to 12-fold higher than the basal level of correlation. The correlation between targets is a measure of the extrinsic noise component of ${CV}_{p_{1}}$ induced by the miRNA titration reactions and gives insights into the possible synchronization of the targets.

Our results show that it is possible to have weakly or highly correlated targets for precise transcriptional programs. The regime of synchronized fluctuations, which is due to the titration-induced retroactivity, is determined by the number of MREs on both targets and is maximal around the threshold for intermediate miRNA repression strengths.

### Interplay between transcriptional activity and miRNA–target interaction strength

Although many mRNAs have more than one MRE for a given miRNA, most of them have just one (see Additional file 1). However, even if there is only one MRE, these targets are typically expressed in multiple copies per cell and, thus, have the potential to titrate away the available miRNA molecules and crosstalk with other targets, if expressed at sufficiently high levels. This suggests testing to see if increasing the number of molecules for a reporter with only one MRE has the same effect on the target competitor as increasing the number of MREs on the same reporter.

**Fig. 6:**
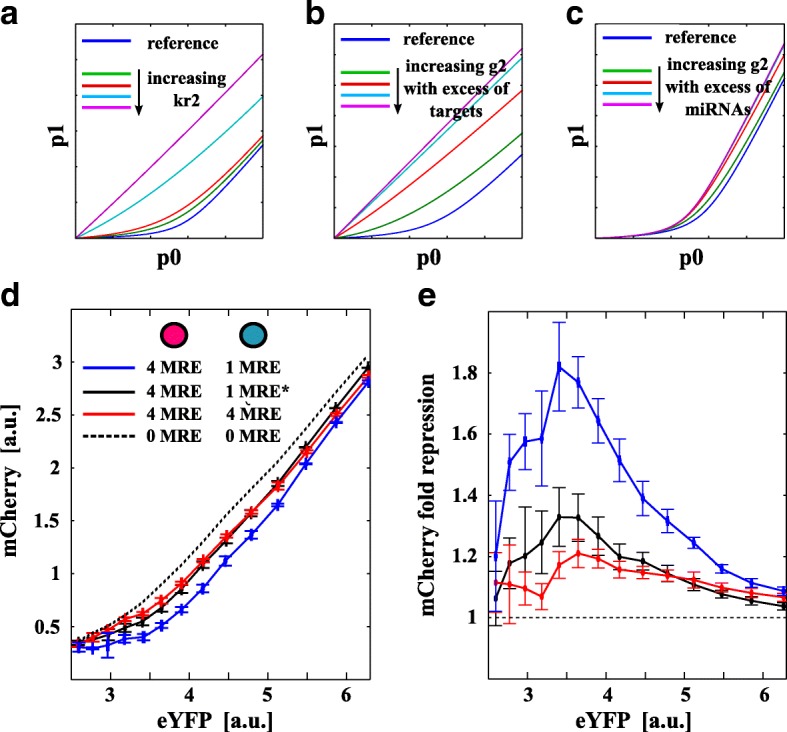
Interplay between transcriptional activity and miRNA–target interaction strength. The figure shows model predictions and experimental results obtained when investigating the effect on one target (say *p*_1_) of the interplay between the second target (say *r*_2_ and, thus, *p*_2_) and the miRNA. The interplay between *r*_2_ and miRNA is tuned both via the transcription rate $k_{r_{2}}$ of *r*_2_ and via the interaction strength *g*_2_ between *r*_2_ and the miRNA. *p*_1_ is plotted against *p*_0_ on **a** increasing the transcription rate $k_{r_{2}}$ of *r*_2_, **b** increasing the interaction strength *g*_2_ between miRNA and *r*_2_ when $k_{r_{2}} > k_{s}$ (excess of targets), and **c** increasing the interaction strength *g*_2_ between miRNA and *r*_2_ when $k_{r_{2}} < k_{s}$ (excess of miRNA). The model prediction for cases depicted in (**a**) and (**b**) are qualitatively very similar. **d** mCherry mean fluorescence (a proxy for *p*_1_ in the model) is plotted against eYFP (a proxy for the constitutive expression *p*_0_ in the model). The *dashed black line* corresponds to the unregulated case while the *blue data points* correspond to the reference case with four MREs on mCherry and one MRE on mCerulean. Either increasing the copy number of mCerulean (a proxy for $k_{r_{2}}$ in the model), *black data points*, or the number of MREs on its sequence (a proxy for *g*_2_ in the model), *red data points*, has the effect of decreasing the amount of miRNA available to target mCherry (which globally increases). **e** Fold-repression with respect to the unregulated case plotted against eYFP. *Error bars* are evaluated on the biological replicates. *a.u.* arbitrary units, *eYFP* enhanced yellow fluorescent protein, *miRNA* microRNA, *MRE* miRNA regulatory element

The model predicts that either an increase (respectively, a decrease) of the transcription rate of target 2 ($k_{r_{2}}$) or of its interaction strength (*g*_2_) causes the same qualitative effect on the system: a decrease (respectively, an increase) of the number of free miRNAs available to bind the first target (*r*_1_). However, the average number of transcripts (〈*r*_1_〉) functionally depends in a different manner on the parameters $k_{r_{2}}$ and *g*_2_. This implies that the two effects, albeit qualitatively similar, are not equivalent (see Fig. 6 a–c).

In particular, increasing $k_{r_{2}}$ has the effect of increasing the basal number of available targets *r*_2_ and simply shifts the *p*_1_ threshold to lower *p*_0_ values. That is, the *p*_1_ curve is shifted towards the left and we see the *p*_1_ curve approaching the unregulated case (see Fig. 6 a). On the other hand, increasing *g*_2_ mimics an increase in the binding efficiency between the target *r*_2_ and the miRNA and results in a decrease in the probability of the miRNA binding to *r*_1_. It is, moreover, possible to define two different regimes, depending on the basal transcription rate of *r*_2_. If the *r*_2_ transcription rate is smaller than the miRNA transcription rate, then on increasing *g*_2_, *p*_1_ will tend to a limiting curve that is different from the unregulated case (see Fig. 6 c). That is, even for a high interaction between miRNA and *r*_2_, there will always be an excess of miRNAs so that *r*_1_ (and *p*_1_) shows a threshold-like behavior. If instead the *r*_2_ transcription rate is bigger than the miRNA transcription rate, then *r*_1_ (and *p*_1_) will tend to the unregulated case when increasing *g*_2_ (see Fig. 6 b) due to an initial target surplus. This last case is qualitatively like increasing the *r*_2_ transcription rate.

We experimentally tested this hypothesis as follows. For a fixed total amount of DNA transfected per cell plate (5 µg), (i) we transfected 1 µg of mCherry reporter with four MREs and 4 µg of mCerulean reporter with one MRE and (ii) we transfected 1 µg of mCherry reporter with four MREs, 1 µg of mCerulean reporter with four MREs, and 3 µg of empty backbone vectors. We then compared the results with the unregulated case (zero MREs on both reporters) and the case with 1 µg of mCherry with four MREs and 1 µg of mCerulean with one MRE. This experiment relies on the assumption that an increase in the quantity of the transfected reporter is a proxy for an increase in the transcription rate of the gene coded in the reporter (i.e., $k_{r_{1,2}}$), while an increase of the number of MREs (i.e., 1, 4, and 7 binding sites) corresponds to an increase of the miRNA–target interaction strength *g*_1,2_.

Our results, shown in Fig. 6 d and e, suggest that increasing the number of MREs on a reporter molecule or increasing the number of reporter molecules with only one MRE have qualitatively similar, although quantitatively different, effects in terms of crosstalk (Fig. 6 e). In particular, they also suggest an excess of targets, since the two curves (red and black) in Fig. 6 d are similar. Since from our target quantification the number of molecules per cell, close to the threshold, is about 150 and the number of mature miR-20a’s is about 1000 molecules per cell, to be in excess of targets the other endogenous miR-20 targets should be active in sequestering the miRNA.

## Discussion and conclusions

**Fig. 7:**
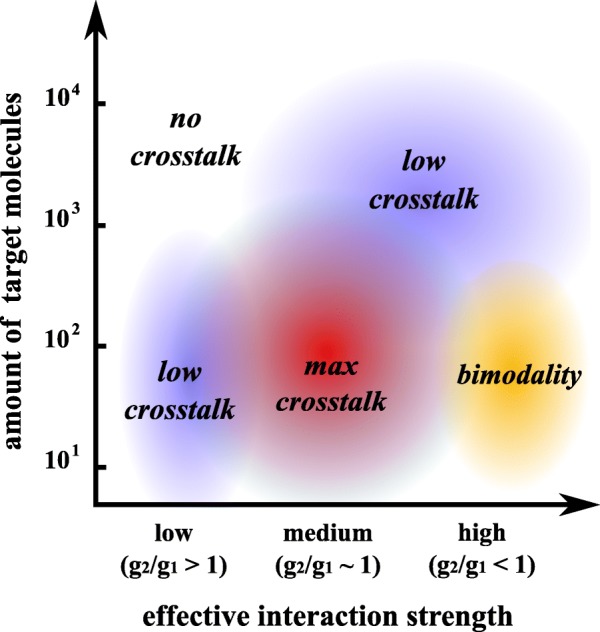
Phase diagram for mCherry (the target product *p*_1_). The figure shows how the crosstalk between targets and bimodality on mCherry behave on varying the effective miRNA interaction strength and the mean numbers of target mRNA molecules. The effective miRNA interaction strength on target *r*_1_ (and, thus, *p*_1_) is measured theoretically through the ratio *g*_2_/*g*_1_ and experimentally with different combinations of miRNA binding sites on both synthetic constructs

Previous studies pointed out the functional relevance of RNA competition for miRNA binding, thus addressing the potential role of RNAs in regulating the distribution of miRNA molecules on their targets [29–34, 43, 44]. In this work, we show, both with stochastic modeling and single-cell experiments that validate the model, that RNAs competing for miRNAs influence their relative fluctuations as well and that this happens in a miRNA-dependent manner. Our results offer a detailed feature map for characterizing the post-transcriptional mRNA cross-regulation (see Fig. 7). Besides the general consistency with previous population-based qualitative results [16] and the agreement with a titration-based mechanism of miRNA–target interaction [17, 20, 21], the stochastic analysis allowed us to characterize curve trends for fluctuations and correlations of two targets of the same miRNA as a function of their expression level. The detailed picture points out that crosstalk between targets is quantitatively relevant only in conditions of intermediate miRNA repression and small amounts of target molecules (of order 10–1000), in agreement with a cell-population-based study by Bosson and coworkers [28].

We stress that the numbers we obtained must be considered more like orders of magnitude than exact numbers, since the way we estimated them, although correct, is indirect [17, 45]. These values are a lower bound on the actual number of molecules per cell since the RNA extraction yield is lower than 100%. Nonetheless, our numbers are compatible with those in [17] and qualitatively in agreement with what we see at the level of fluorescence (i.e., the amount of exogenous RNAs increases on increasing the fluorescence of the eYFP reporter). This finding is in contrast with that in [35] where, with a similar experimental design, the authors observed that the exogenous targets are expressed as the most expressed endogenous genes. Despite that both our study and [35] used the HEK 293 cell line, the evaluated endogenous miRNAs are different: miR-20a in our case and miR-21 in [35]. According to [38], miR-20a is the most expressed miRNA in HEK 293 whereas miR-21 is not even in the list of the 20 most expressed miRNAs in that cell line. This implies that if the relative stoichiometry between miRNA and targets matters with respect to the effectiveness of competition, the discrepancy with what was observed in [35] may be related to the endogenous amount of miR-21. Indeed, we showed that the effectiveness of the competition is limited to particular stoichiometric conditions and that these conditions are achieved near the threshold produced by the miRNA–target titration reactions. Although cross-regulation between targets is not zero even for a high level of target fluorescence (high mRNA expression), the region of maximal sensitivity of the system is limited.

The miRNA–target titration reactions introduce correlations among the intrinsic noise of one target and the extrinsic noise of the miRNA and in turn the extrinsic noise of the other miRNA target. The model predicts that this phenomenon of noise coupling, first introduced in [41] as a consequence of binding reactions, is large near the threshold, when the amount of free miRNA is comparable to the number of free targets. The idea that binding reactions introduce correlations is defined as retroactivity (see [46] for a review) and described at the mean field level in [47]. Our results are a vivid manifestation of this retroactivity phenomenon, which per se can drastically affect noise transmission in cellular networks and eventually impede a modular description of biochemical networks [41]. However, when the retroactivity is high, two or more targets may be highly cross-correlated, thus our results suggest that optimal levels of expression of genes and of miRNAs with respect to maximizing retroactivity may control the relative fluctuations of targets that have to interact or bind in complexes with a precise stoichiometry [22].

On the other hand, we found that strong miRNA repression together with low target crosstalk is sufficient to induce bimodality, i.e., the appearance of two distinct populations of cells with low and high target expression states. Bimodally expressed genes are found in different contexts, ranging from breast cancer [48] to immune cells [49], and usually each mode of the bimodal distribution corresponds to a different physiological condition (for example, a normal or a disease state). Our results suggest that if the bimodally expressed gene is a miRNA target, the system could be locked in one of the two states, changing the miRNA–target interaction strength through the expression of other competitors.

Our experimental strategy allowed us to study the interplay between MRE multiplicity and the effect of differentially expressing one of the two targets. The stochastic model predicts, and our results confirm, that the effect of increasing the transcription rate of one of the two targets reduces the share of free miRNAs available to repress the other target. Qualitatively, the same effect could be achieved by increasing the miRNA–target interaction strength of one of the two targets. Although from a purely mathematical point of view the dependence of the average number of proteins of the first target on the transcription rate and on the miRNA–target interaction strength of the second one are different, there are ranges of parameter values for which this distinction is not so sharp.

According to Sætrom and colleagues, two miRNA molecules can cooperate in the repression of their target when seed binding sites are 13–35 nucleotides apart [50], a phenomenon that has also been studied theoretically [51, 52]. In contrast, MREs very near to each other may result in the interference of miRNA binding. Experimentally, we modulated the interaction strength through the number of MREs on the constructs. Our constructs have bulged miR-20a sites separated by four-nucleotide-long spacers [17] and the seed binding sites are 18 nucleotides from each other, which should imply the cooperative miRNA repression of the targets. However, there is no significant difference in the repression of mCherry with *N*=4 and *N*=7 MREs if there are no MREs on mCerulean (Fig. 2 a) and there is little shift in the mCherry fluorescence when the number of MREs on mCerulean increased from one to four (Fig. 2 b). A possible key to interpreting our results is that upon increasing the number of MREs per construct what is really increasing is the probability of miRNA binding to the target and not the number of miRNAs simultaneously bound to a single target. However, as recently shown in [53], the very mechanism of the miRNA–target interaction is not yet completely understood, and we expect new findings in the near future that will put our mathematical modeling on more solid ground.

It is tempting to speculate that gene expression thresholding could be an important feature of cell fate decisions. However, while titrative regulatory mechanisms of miRNAs may easily switch whole gene networks on or off depending on the relative stoichiometry of miRNAs and targets, the scope of this mechanism has to be determined case by case. Indeed, theoretically, there is in principle no limit on the number of genes that can be cross-regulated by one gene or via the expression of a common miRNA [20] (see Additional file 1), given that it is just a matter of parameters to tune to the appropriate value (for example, the transcription rate of one target or miRNA). In physiological conditions, however, where there is no fuel turnover, the number of genes involved in the crosstalk may be limited and case specific.

Taken together, our results suggest as well that cross-regulation is relevant for molecules present in small amounts. Molecular species physiologically present in the order of 10–1000 molecules per cell, such as transcription factors or signaling molecules [37, 54], are more likely to be affected by cross-regulation. Although our experimental setting is artificial, it provides a deep exploration of the parameter space. A physiological system of miRNAs and targets could indeed experience only a small subset of the features so far described, rendering the characterization of crosstalk difficult. Without needing to over-express any endogenous gene, we were instead able to mimic the variation of relevant model parameters (miRNA and target transcription rates and miRNA–target interaction strength). Our phase diagram (Fig. 7) links quantitative measurements (effective miRNA repression and number of mRNA molecules) with model parameters, suggesting the possibility of moving around in the phenotype space tuning quantities such as the accessibility of binding sites or the affinity between miRNA and targets.

The functional importance of miRNA-mediated target cross-regulation has been shown in a number of cases, both diseased and physiological. For example, the pseudogene PTENP1 regulates the expression of the tumor suppressor PTEN in a miRNA-dependent manner, eventually modulating cancer cell growth [43, 55]. Our findings suggest the possibility that when PTENP1 crosstalks with PTEN, their corresponding RNAs are close to the maximum-crosstalk region of the phase diagram. Tuning the miRNA–PTEN interaction strength through the expression of the miRNA-competitor PTENP1 would then move the system closer to the bimodality region, with the two modes of the distribution being the cancer and the normal cells. At this point, the cell population can switch from one subpopulation to the other and the two competitors are highly coupled. Subsequent downregulation of PTENP1 would then lock the system in one of the two states and move it toward the low-crosstalk region of the phase diagram, characterized by a monomodal distribution of the cell population. Another relevant case involves the long non-coding RNA linc-MD1, which competes for binding miRNAs with the two transcription factors MAML1 and MEF2C. Thus, it regulates the differentiation of myoblasts in normal muscle development [44]. As a last example, the 3^′^ UTR of CD44 competes for miRNA binding with the mRNA of CDC42, whose corresponding protein regulates the cell cycle [56]. We think that our findings give theoretical support and valuable tools for making significant progress in the understanding of these and other miRNA-mediated target cross-interactions.

## Methods

A detailed description of the modelling and data analysis procedures is available in Additional file 1 (Supplementary Material).

### Reporter plasmid construction

The set of fluorescent reporters coding for eYFP and mCherry was obtained from Addgene (*#*31463, *#*31464, *#*31465, and *#*31466, deposited by Phil Sharp Lab) and are the same as those used in [17]. The second set of fluorescent reporters were cloned into pBI-CMV1 (Clontech). A nuclear localization sequence (NLS) (ATGGGCCCTAAAAAGAAGCGTAAAGTC) was appended to mCerulean-N1 (Addgene *#*27795, deposited by Steven Vogel Lab [57]) by PCR and then inserted into the main vector with ClaI and BamHI. mKOrange-NLS (Addgene *#*37346, deposited by Connie Cepko Lab [58]) was cloned into the vector using EcoRI blunt and BamHI. miR-20a regulatory elements were appended to the 3^′^ UTR of mCerulean with the same strategy applied in [17]: the *N*=1 bulged miR-20 binding site (TACCTGCACTCGCGCACTTTA) was appended by PCR and for both constructs, CCGG spacers separate subsequent miR-20a regulatory elements.

### Transient transfections

We performed two different methods of transfection, with Lipofectamine (data in Figs. 2, 3 and 4 in the main text and in Additional file 1: Figure S3) and with CaCl_2_ (data in Additional file 1: Figures S1 and S2). Our results are independent of the method of transfection.

**Lipofectamine transfection method** HEK 293 TeT-Off cells (Clontech) below passage 6 were plated in G418 (Gibco) 200 µg/ml media in six-well dishes the day before transfection. Reporter plasmids were transfected with Lipofectamine 2000 (Invitrogen) following the manufacturer’s specifications. miR-20a pre and negative controls (Ambion) were cotransfected at the indicated concentrations. The media was changed 24 h after transfection. Assays were performed 48 h after transfection.

**CaCl**_**2**_ **transfection method** HEK 293 TeT-Off cells (Clontech) below passage 6 were plated in 100×20 mm (Falcon BD) dishes the day before transfection. The cells were transfected using CaCl_2_ protocols [59]. The media was changed 24 h after transfection. Assays were performed 48 h after transfection.

### Flow cytometry

Cells were harvested 48 h after transfection (cell confluency ∼90 %) and run on a CyanADP (Beckman Coulter) flow cytometer. For each sample, at least 0.5×10^6^ cells were acquired. The raw FACS data were analyzed with the Summit3.1 software (Beckman Coulter) to gate cells according to their forward and side scatter profiles and to define the intensity of fluorescent signals emitted by the four reporters in each cell. These values were normalized for background fluorescence by subtracting the mean plus two standard deviations of the fluorescent signal measured in the untransfected control cells. The data were then binned according to their eYFP values.

### Data processing

For each cell, we collected the raw fluorescent intensities and then we subtracted the background fluorescence levels estimated from non-transfected cell reads. The background-corrected data were then binned into consecutive equally spaced intervals of eYFP intensities. Each bin contains a subpopulation of the order of 10^4^ cells. Eventually, each bin is characterized by the mean fluorescent intensity, by the coefficient of variation for each fluorophore, and by the Pearson correlation coefficient between mCherry and mCerulean (computed for each subpopulation). The same procedure was then applied to the three different biological replicates. The final values we report in data plots (Figs. 2 a, b, 3 a and 4 a, b in the main text and Additional file 1: Figures S1 and S2) are the mean of these measurements over the biological replicates for each bin, and the error bar is their standard deviation. It is probably worth stressing that the error bar computed in this way is a proxy for the fluctuation of the mean over the biological replicates rather than the data dispersion, the latter being a factor $\sqrt {N_{\text {cib}}}$ larger than the former, where *N*_cib_ is the number of cells in a bin (see Additional file 1: Figure S4). The error bars for Figs. 2 c–e and 3 b are instead computed with a standard jackknife procedure.

### Fluorescence-activated cell sorting

Cells were transfected with the *N*=4 eYFP-mCherry and *N*=1 mkOrange-mCerulean reporters with and without pre-miR-20a 100 nM (Ambion). 48 h after transfection, three cell populations were sorted according to their eYFP fluorescence (low, medium, or high YFP expression) using a BD FACS Aria III (Becton Dickinson) cell sorter. Cell pellets were washed and snap-frozen before RNA isolation.

### Empirical observables and Pearson correlation coefficient ratio

We defined the empirical average of a given observable *O* over an ensemble of cells as $\langle O \rangle = \sum _{i\in \text {cell}} O_{i}/N_{\text {cell}}$. The Pearson ratio is defined as the ratio of the Pearson correlation coefficient (*ρ*_*x,y*_=(〈*xy*〉−〈*x*〉〈*y*〉)/*σ*_*x*_ *σ*_*y*_) between mCherry and mCerulean with a given combination of MREs to the same measure in the absence of MREs. We evaluated the ratio for each eYFP bin (below, around, and above the threshold) for at least three different biological replicates. We then estimated the *p* values of each ratio with respect to the distributions having as standard deviation the error on the biological replicates and as mean values the Pearson ratio for mCherry and mCerulean with *N*=0 MRE for the three eYFP intervals.

## Additional file

Additional file 1 Supplementary Material. Detailed description of the modeling and data analysis procedures. (PDF 1106 kb)
