# Supplementary material for: RNAs competing for microRNAs mutually influence their fluctuations in a highly non-linear microRNA-dependent manner in single cells
Source: Genome Biol. 2017 Feb 20;18:37. doi: 10.1186/s13059-017-1162-x (PMC5319025; doi:10.1186/s13059-017-1162-x)
Supplement: Supplementary file 1 — Supplementary Material. Detailed description of the modeling and data analysis procedures. (PDF 1106 kb) [file 13059_2017_1162_MOESM1_ESM.pdf]

RNAs competing for microRNAs mutually influence their fluctuations in a highly non-linear microRNA-dependent manner in single cells - Supplementary Material

Carla Bosia, Francesco Sgrò, Laura Conti, Carlo Baldassi, Davide Brusa, Federica Cavallo, Ferdinando Di Cunto, Emilia Turco, Andrea Pagnani and Riccardo Zecchina

November 11, 2016

Contents

1 Absolute quantification of mean number of exogenous molecules per cell 2
1.1 Cell sorting . . . 2
1.2 RNA extraction and qRT-PCR . . . 2
1.3 Standard curves . . . 2
1.4 Internal control and C_T correction . . . 3
1.5 Final quantification . . . 3

2 Absolute quantification of mean number of miR-20a molecules per cell 5

3 Stochastic model of molecular titration and crosstalk 6
3.1 Model definition . . . 6
3.2 Independent molecular-species approximation . . . 7
3.3 Gaussian Approximation . . . 8
3.4 A network perspective . . . 9

4 Distribution of targets with multiple MREs 11

5 Supplemental figures 11

1 Absolute quantification of mean number of exogenous molecules per cell

In the following we describe step by step the procedure we followed to obtain the estimate of the mean number of exogenous RNA molecules per cell in the cases with and without pre-miR20a. The entire procedure was repeated three times, for three different biological replicates (that is, three different cotransfection experiments with and without pre-miR20a). In Table 1 in the main text we reported the means and errors over these biological replicates.

1.1 Cell sorting

We sorted the cotransfected cells into three groups depending on the level of eYFP fluorescence. To define the three intervals of fluorescence we identified the threshold at the cell sorter plotting mCherry vs eYFP for the cells cotransfected with the pre-miR20a. We then sorted the cells for eYFP below threshold (i.e. eYFP-low sample), around threshold (i.e. eYFP-medium sample) and above threshold (eYFP-high sample), see Figure S5 for an example. For the same eYFP intervals, we sorted as well the cells without pre-miR20a.

1.2 RNA extraction and qRT-PCR

Total RNA was extracted from each sorted cell subpopulation using Trizol reagent (Ambion Life Technologies, USA) in combination with Pure Link RNA Mini Kit (Ambion). $1\mu g$ of total RNA was treated with RQ1 DNAse (Promega) and reverse transcribed, after heat-inactivation of the enzyme, using M-MLV reverse transcriptase and random primers (Life Technologies). Quantitative RT-PCR was performed on a 7300 Real Time PCR System (Applied Biosystems) using specific primers for eYFP, mCherry, mCerulean and mKOrange. 18S probe (Life Technologies) was used as internal loading control. In particular, qRT-PCR was performed (i) on cDNA of the sorted cotransfected cells with primers for the exogenous targets and for the endogenous ribosomal RNA 18S and (ii) on DNA standards spiked into untransfected cell cDNAs (same protocol used in [1]). The DNA standards were different dilutions of amplicons for the exogenous targets (eYFP, mCherry, etc...), each with its corresponding pair of primers. The use of these standards allowed the definition of a calibration curve for each fluorophore, directly linking threshold cycles and mean number of molecules per cell [2].

1.3 Standard curves

DNA standards were used at amounts ranging from $0.5\,pg$ to $0.005\,fg$ per reaction. For each pair of primers (and cDNA sample) we had three technical replicates (so, three technical replicates for the eYFP-low cDNA sample with mCherry primers, three technical replicates for the eYFP-low cDNA sample with eYFP primers, etc...). The different dilutions of amplicons allowed the definition of a standard curve for each

amplicon that directly links the mass pre-PCR of the amplicon and the threshold cycle (C_T) of the qRT-PCR reactions [2]. The C_T were in the exponential phase of PCR amplification (see [2, 3]). In Figure S6 we show an example of standard curve for the mCherry amplicon.

1.4 Internal control and C_T correction

We used the 18S rRNA amplification as an internal loading control, to correct for the possible differences in total RNA input and retro-transcription efficiency from one cDNA sample to the other. As previously described in [3], we first checked the stability of this control in our setting by comparing the mean 18S C_T (as 2^{-C_T}) between the experiment without pre-miR20a (1.8719×10^{-5}) and the experiment with pre-miR20a (1.7374×10^{-5}). The fold-change between the internal controls is $0.93 \sim 1$, thus confirming that the transfection of pre-miR20a does not change the expression of the 18S. For each experiment we chose the lowest 18S C_T as a reference. We then evaluated the difference between the lowest 18S C_T and the other 18S C_Ts. Such differences are the correction terms we add to the C_Ts ("corrected C_T") of the samples we want to quantify (with primers for mCherry, eYFP, etc...). The choice of the lowest 18S C_T is to avoid underestimating the average number of molecules/cell. However, using the highest or the mean among all the 18S C_Ts did not change significantly the order of magnitude of the results, indicating homogeneous loading and retro-transcription efficiency across our samples.

1.5 Final quantification

After obtaining corrected C_Ts for all the samples, we use the calibration curves to know the corresponding pre-PCR mass for the amplicons [2, 1]. This is the mass corresponding to $1\mu g$ of RNA. Since we know the total mass of RNA extracted, the molecular weight of the amplicons and the number of cells per sample, it is then straightforward to evaluate the mean amount of exogenous RNA molecules per cell:

$$\text{mean exogenous RNA per cell} = \frac{m \times d \times \mu \times MW \times N_A}{N} , \qquad (1)$$

where m is the RNA pre-PCR mass in $1\mu g$, d is the dilution factor of the cDNA sample, μ are the corrected total μg of extracted RNA, MW is the molecular weight of the amplicon (the product of all these quantities is the number of moles of that particular amplicon in the sample), N_A is the Avogadro number and N is the number of cells in the original sorted subpopulation of cells. μ is defined as $\mu = C \times \text{total RNA}\,\mu g$, where C is the ratio

$$\frac{(\text{total RNA}\,\mu g/N)_{\text{max}}}{\text{total RNA}\,\mu g/N} .$$

C is thus the ratio between the mean amount of total RNA per cell in the sample where this quantity is the highest and the other samples. This correction takes into account possible technical pipetting errors (anyhow small, since $C \sim 1$ for all the samples). Here below in Table 1 we show as example of the estimate of the mCherry

mean amount of molecules per cell in the "eYFP medium" sample for one biological replicate (see also Figure S6). Consider however, that mean values and standard deviations presented in Table 1 in the main text are computed over the different biological replicates (that is, over different co-transfection experiments of the same constructs). Indeed for each experiment, in which the cells have been sorted in the

Table 1: Evaluation of mCherry mean amount of molecules per cell for one biological replicate (three technical replicates).

corrected C_T	pre-PCR mass	# moles	# molec. per cell
18.45	1.64 $\times 10^{-13}$ g	7.83 $\times 10^{-17}$	64
18.41	1.69 $\times 10^{-13}$ g	8.04 $\times 10^{-17}$	66
18.52	1.57 $\times 10^{-13}$ g	7.49 $\times 10^{-17}$	61
mCherry molecular weight	139260 Da		
number of cells	736181		
total RNA mass in the sample	6420 $\times 10^{-9}$ g		
RNA correcting factor C	1.03		

intervals "eYFP low", "eYFP medium" and "eYFP high", we have three technical replicates per sample. The technical errors, as can be noticed from the small dispersion of the three estimated number of molecules per cell in Table 1 in SI (here above), is much smaller than the biological one (that is, the error we reported in Table 1 of the main text). In the following we report a summary table of the exogenous transcripts quantification for the biological replicates (mediated over the technical ones).

Table 2: Biological replicates for exogenous transcripts quantification.

eYFP high		
exogenous transcripts	Cotransfection	Cotransfection + pre-miR-20a
eYFP high	310, 5965, 2682	365, 308
eYFP medium	125, 123, 813	114, 150, 44
eYFP low	100, 16	29, 20, 73
mCherry high	93, 821, 358	137, 34, 445
mCherry medium	64, 110, 164	8, 41, 14
mCherry low	53, 16	6, 4, 21
mCerulean high	37, 327, 259	70, 111, 602
mCerulean medium	14, 47, 120	32, 66, 9
mCerulean low	5, 7	5, 10, 9

2 Absolute quantification of mean number of miR-20a molecules per cell

To quantify the mean number of mature endogenous miR-20a molecules per cell we adopted a spike-in procedure following the guidelines described in [4]. In particular, we lysed independent samples containing a fixed amount of cells ($\sim 2 \times 10^6$) with QIAzol lysis reagent (QIAGEN) and added to lysates increasing amounts of synthetic mature miR-20a (UAAAGUGCUUAUAGUGCAGGUAG). Total RNA was then extracted from each sample using miRNeasy mini kit (QIAGEN). Using both TaqMan MicroRNA Assay for hsa-miR- 20a (Thermofisher) and RNU44 TaqMan MicroRNA control Assay (Thermofisher), we reverse transcribed $10ng$ of total RNA per sample and performed quantitative RT-PCR on a 7300 Real Time PCR System (Applied Biosystems). The RNU44 control was used as internal loading control to correct for the possible differences in total RNA input and retro-transcription efficiency from one cDNA sample to the other. This control was used exactly as we did with S18 for the quantification of exogenous target molecules. The corrected C_Ts for all the 10 samples were then directly linked to the mean amount of miR-20a molecules per cell (see Figure S7). The changing in slope of the resulting curve corresponds to the amount of endogenous miR-20a molecules per cell (1250 molecules per cell in our case). We performed as well qRT-PCR for two samples of cell, one transfected with 100 nM of pre-miR20a 48 hours before the RNA extraction and the other untransfected. We then used the untransfected sample as reference to measure the $\delta\delta C_T$ between its C_T and the C_T of the pre-miR transfected sample. The pre-miR transfected sample shows a 163 fold increase in the mature miR-20a with respect to the untransfected one.

Cotransfection experiments in HeLa TeT-ON cell line

To show that our results are unbiased with respect to the cell line we repeated part of the experiments in HeLa cells. We performed cotransfection experiments with and without 100 nM of pre-miR20a for the unregulated case (0 MRE on both mCherry and mCerulean) and for reporter with 4 MRE on mCherry combined with mCerulean with 0,1,4,7 MRE.

Cells were plated in 6 well multiwells and induced with doxicycline 24 hours before transfection. We used Effectene (QIAGEN) as transfection reagent and measured the florescence on a CyanADP (Beckman Coulter) flow cytometer.

Results, shown in Figure S8 and S9, summarize what we found in HEK 293 Tet-OFF cell line. That is, microRNA-mediated crossregulation is context dependent but there is an optimal range of parameters for which crossregulation is maximal among targets. In particular, it arises (i) at the level of mean protein concentrations (see the threshold effect in Figure S8a, the modulation of the threshold when increasing the miR20a pool via premiR transfection in Figure S8b and the fold repression in both cases in Figure S8c); (ii) at the level of fluctuations (see Figure S9ab, where the mCherry total noise is modulated via the expression of mCerulean with an increasing number of MRE) and correlations (the ratio between the Pearson correlation coefficient in presence of

miRNA mediated regulation and the unregulated case is maximal for eYFP values close to the threshold, see Figure S9c).

3 Stochastic model of molecular titration and crosstalk

3.1 Model definition

We describe with a stochastic model the miRNA-target interactions. The system can be described by 5 interacting variables (1 miRNA, 2 mRNAs, 2 proteins) indicated respectively as s, r_1, r_2, p_1, p_2, which represent the (integer) copy number of molecules present in the cell at any given time t. Using this notation, the probability P of finding in a cell exactly s, r_1, r_2, p_1, p_2 molecules at any time t is governed by the following master equation:

$$
\begin{aligned}
\partial_t P \;=\;& \sum_{i=1}^{2}\left[k_{r_i}(P_{r_i-1}-P)+k_{p_i}r_i(P_{p_i-1}-P)\right]+k_s(P_{s-1}-P)\\
+\;& \sum_{i=1}^{2}\{g_{r_i}[(r_i+1)P_{r_i+1}-r_iP]+g_{p_i}[(p_i+1)P_{p_i+1}-p_iP]\}+g_s[(s+1)P_{s+1}-sP]\\
+\;& \alpha\sum_{i=1}^{2}g_i[(r_i+1)(s+1)P_{r_i+1,s+1}-r_isP]\\
+\;& (1-\alpha)\sum_{i=1}^{2}g_is[(r_i+1)P_{r_i+1}-r_iP] \qquad (2)
\end{aligned}
$$

where $P := P_{r_1,r_2,p_1,p_2,s}$ and, for example P_{p_2+1} is a short hand notation for $P_{r_1,r_2,p_1,p_2+1,s}$. In Eq. (2) k_{r_i}, k_s and k_{p_i} (with $i = 1, 2$) are the transcription rates of mRNAs r_i and miRNA s and the translation rates for proteins p_i respectively. g_{r_i}, g_s and g_{p_i} (with $i = 1, 2$) are their degradation rates. g_i (with $i = 1, 2$) are the effective association rates for the miRNA s and the mRNA r_i. Finally the parameter α measures the catalyticity of the interaction, *i.e.* the fraction of miRNA molecules that are recycled after the interaction with their targets. This master equation is not amenable for analytic solutions and approximate methods have been proposed [5, 6] to obtain accurate quantitative predictions. Following previous work [6] we obtained the approximated expression for mean values, standard deviations and Pearson correlation coefficients (sketched in Figure 1b-d in the main text), which we describe in the next paragraphs.

The model can be easily generalized for a system of M miRNAs $s_1, \ldots, s_M$, N mRNAs $r_1, \ldots, r_N$, and N proteins $p_1, \ldots, p_N$. In the following, we will assume that each mRNA is coding for a distinct protein. However, within this framework, we could easily describe, for instance, non-coding RNAs. In other terms we are not bounded to have the number of proteins N equal to that of the different mRNA species. This generalized model reads:

$$
\begin{aligned}
\partial_t P &= \sum_{i=1}^{N} [k_{r_i}(P_{r_i-1} - P) + k_{p_i} r_i (P_{p_i-1} - P)] + \sum_{i=1}^{M} k_{s_i}(P_{s_i-1} - P) \\
&+ \sum_{i=1}^{N} \{g_{r_i}[(r_i+1)P_{r_i+1} - r_i P] + g_{p_i}[(p_i+1)P_{p_i+1} - p_i P]\} \\
&+ \sum_{i=1}^{M} g_{s_i}[(s_i+1)P_{s_i+1} - s_i P] \\
&+ \alpha \sum_{i=1}^{M}\sum_{j=1}^{N} g_{ij}[(r_i+1)(s_j+1)P_{r_i+1,s_j+1} - r_i s_j P] \\
&+ (1-\alpha)\sum_{i=1}^{N}\sum_{j=1}^{M} g_{ij} s_j [(r_i+1)P_{r_i+1} - r_i P] \qquad (3)
\end{aligned}
$$

Provided that the values of all parameters of the model (e.g. rates of transcription, degradation, etc.) are known, it is in principle possible to deal with a large number of chemical species, both in terms of stochastic simulation *a la* Gillespie, or in term of controlled mathematical approximations, that we are going to outline in the following. A proof of the principle of how the model behaves in the more realistic case of 10 mRNA targets regulated by 10 microRNa in blocks overlapping blocks, has been already extensively discussed (see in particular Figure 4 in [6] and the related discussion in the main text). In the following we will also give a network about the competitive titration mechanism at large scale.

In the following analysis, unless otherwise stated, we will just consider the specific case $N = 2, M = 1$ which provides a reasonable model for experimental setup, but, again, more complicated mRNA-microRNA interaction architectures could be analyzed using the same techniques.

3.2 Independent molecular-species approximation

As long as one is interested in mean values of the observables at steady state, a good approximation is the so-called independent molecular species approximation, also known as mean-field approximation which amounts to assume that the multivariate probability distribution P is factorized among the different chemical species:

$$
P^{\text{ind}}(r_1, r_2, p_1, p_2, s) := P_{r_1}(r_1)P_{r_2}(r_2)P_{p_1}(p_1)P_{p_2}(p_1)P_s(s) \ . \qquad (4)
$$

Plugging this factorized functional form into Eq. (2), and computing the first moments at steady state (*i.e.* in the limit $t \to \infty$), one obtains a system of second order equations in the five variables which is easily solved numerically for any value of the model parameters. The main limitation of the factorized ansatz in Eq. (4) is that, although giving a fairly accurate prediction of the mean values of the different chemical species across a wide range of parameters, the very simple structural form of Eq. (4) cannot

predict their statistical correlations. For example the Pearson correlation coefficients under the independent chemical species approximation are always zero by definition. We used the independent chemical species approximation to fit the model parameters through the following functional law:

$$f(x|\lambda, \theta, m) = m[(x - \lambda - \theta) + \sqrt{(x - \lambda - \theta)^2 + 4\lambda x}] \quad (5)$$

where x is the transcriptional activity (in terms of eYFP fluorescent value), $\theta = k_S/\alpha$ and $\lambda = g_R g_S/(\alpha g)$, as it can be deduced by the detailed derivation in [6]. The further parameter m is introduced here to take into account the fact that the intrinsic brightness depends on the fluorescent protein type in such a way that, for instance, even for the construct with no miRNA binding sites the straight line (c.f. Figure 2 in the main text) has a slope different than one. It can be shown perturbatively that in the limit of interaction strengths of the same order of magnitude, the same functional form displayed in equation (5) holds also in the multiple target case. From the functional form of Eq. (5), it is also clear that $f(0|\lambda, \theta, m) = 0$ for all value of the parameters. We thus manually rescaled the fluorescent values so that each curve passes through the origin. Interestingly the same constant values for the rescaling hold for any combination of the constructs.

Of the three fit parameters defined in Eq. (5), the last parameter m has no biological interpretation, and accounts only for the different intrinsic brightness of the different fluorescent proteins. The parameter $\theta = k_S/\alpha$ is the ratio between miR20a transcription rate and the turnover α. Unfortunately for both parameters very little is known in the context of our experiment (293-HEK cell line) and the result of our fit is given in terms of rescaled fluorescent units, rather than in terms of rate of transcription activity. The second parameter $\lambda = g_R g_S/(\alpha g)$, with $g = k^+\gamma/(k^- + \gamma)$, involves a combination of biochemical parameters for miRNA/mRNA complex formation (association and dissociation rates k^+, k^- and degradation rate γ) which are known only for some miRNAs in-vitro. Moreover, the validity of these estimates in-vivo is often debated.

3.3 Gaussian Approximation

To overcome the above mentioned limitations and to take under control correlations across chemical species, a very simple yet accurate approximation scheme is the so-called Gaussian one [6]. Note that, following this approximation scheme, copy numbers will not be bound to be integer numbers as it was for the master equation defined in Eq. (2). As we will see in the following, and has already been extensively discussed in [6], this does not affect the good quality of the approximation. Let us denote with $\vec{X}$ the five-dimensional vector of components r_1, r_2, p_1, p_2, s respectively. We can thus make the following multivariate Gaussian ansatz for the probability distribution function of $\vec{X}$:

$$P^{\text{Gauss}}(\vec{X}) := \frac{1}{\sqrt{(2\pi)^5 \det C}} \exp\left[-\frac{(\vec{X} - \vec{\mu})^T C (\vec{X} - \vec{\mu})}{2}\right] \quad , \quad (6)$$

which in our 5−dimensional case depends on 20 parameters: 5 numbers specify the mean $\vec{\mu}$ and 15 the covariance matrix C (which is symmetric). The key property that makes Eq. (2) very difficult to solve analytically is that, as shown in detail in [6], it generates a whole hierarchy of moments such that the lower moments are expressed in terms of higher order moments and no moment-closure scheme can be utilized. Multivariate Gauss distributions, on the other hand, have the useful property that all moments can be expressed as a linear combination of just the first and the second moments. As an illustrative example, defining $\mu_i = E(X_i)$ and $E(X_i X_j) - E(X_i)E(X_j)$ for $i \neq j$, we could consider the generic third moment of the distribution defined in Eq. (6) $E(X_i, X_j, X_k) = C_{ij}\mu_k + C_{ik}\mu_j + C_{jk}\mu_i$ for $i \neq j \neq k$, where C_{xy} is the x-row y-column element of the covariance matrix C.

A systematic procedure to compute $\vec{\mu}$ and C requires to define the time dependent moment generating function:

$$F_t(\vec{z}) := \prod_{i=1}^{5} z_i^{X_i} P_t(\vec{X}) \tag{7}$$

Plugging the above equation in the master equation we get the following second order partial differential equation:

$$\partial_t F_t(\vec{z}) = \mathcal{H}(\vec{z}) F_t(\vec{z}) \tag{8}$$

The moment generating function has the following properties:

$$\begin{aligned} F(\vec{z} = 1, q = 1) &= 1 \,, \\ \partial_{z_i} F|_{\vec{z}=1,q=1} &= \langle X_i \rangle \,, \\ \partial^2_{z_i} F|_{\vec{z}=1,q=1} &= \langle X_i^2 \rangle - \langle X_i \rangle \,, \\ \partial^2_{z_i,z_j} F|_{\vec{z}=1,q=1} &= \langle X_i X_j \rangle \,. \end{aligned} \tag{9}$$

By inserting the previous definitions and imposing the Gaussian marginalization conditions mentioned above, we obtain at the steady state a system of 20 equations in 20 unknown that we can numerically solve to get the values for $\vec{\mu}$ and C. As already shown in [6], this approximation turns out to reproduce fairly accurately both noise (in terms of coefficient of variation CV) of single targets and Pearson correlation coefficient between targets, when compared with the numerical values obtained through Gillespie algorithm.

3.4 A network perspective

Considering that typically a given miRNA might have hundreds endogenous targets, a puzzling issue of miRNA mediated crosstalk is to what extent a reasonable fold change (say a two to seven fold increase) of one of the targets could produce an observable effect on any other target. Such a situation is observed in our experiment where the transcripts coded by our exogenous constructs are actually competing with the rest

of the endogenous targets. This issue was already quantitatively addressed in [6], and here we just report the main conclusions.

Let us consider a system where one miRNA s is interacting with 100 targets $t_1, \ldots, t_{100}$. We are interested in the effect on the system at steady-state of a k-fold increase of transcription rate of target t_1 on a randomly chosen target, say t_2. The overall scenario is dictated by the relative stoichiometry of the system, and in particular on the share of free miRNA s (*i.e.* number of miRNA molecules which are not bound to their targets). Three scenarios are in principle possible:

(i) The number of free targets is larger then the total number of miRNA ($s = 0$, all miRNAs are bound to a target and there is a part of the targets which is free).

(ii) All targets t_i are bound to a miRNA but there is still a share of free miRNA ($s \gg 0$).

(iii) A marginal quasi-equimolarity condition where the number of targets equals on average the total number of miRNAs ($s \sim 0$).

Without resorting to mathematical modeling, one can understand that, for different reasons, under scenarios (i) and (ii) very little or null crosstalk between targets is to be expected.

In the first case (excess of targets) an increase in concentration of the first target will indeed be neutral to the second as, being all miRNAs entirely sequestered, the increase will not able to release further miRNAs from the second one. This simple intuition can be made more quantitative by observing Figure S10, where we display the mean number of molecules for t_1, t_2 and s as a function of k_{r_1} (i.e. the transcription rate of target 1) obtained from direct integration of Eq.(2). One can see how the value of t_2 is saturating to its constitutive expression value (i.e. the value of the transcript in the absence of miRNAs). Given the convexity of the two curves, it is also clear that, well above threshold, an increase δ_1 on t_1 will induce a much smaller δ_2 on t_2.

In the second case (excess of miRNAs), the system is so repressed that, well below threshold, any fluctuations of any of the targets would be immediately down-regulated by the excess amount of miRNAs. Note also that, in the case of our experiment, the (unbounded) share of target t_1 and t_2 can be considered as a proxy for the quantity of the fluorescent protein they code for. Under condition (ii), as shown in the leftmost part of Figure S10, virtually no protein can be translated, and therefore no crosstalk effect could be observed.

Things become more interesting in the quasi-equimolarity condition (iii), where due to the strong non-linear behavior of our system at threshold, crosstalk becomes indeed possible. As shown in [6] it turns out that a 2-fold increase in the transcription rate of t_1 causes a 1.3-fold increase on t_2, whereas a 7-fold increase on t_1 would cause a 4.5-fold increase on t_2 (but of course the same would be true for any other target).

4 Distribution of targets with multiple MREs

In our two reporters system, we have considered, in analogy with [1], different numbers of MREs (0,1,4,7) for the same miRNA family as a proxy of the miRNA-target interaction strength (encoded in the rates g_1, g_2 in our model). From now on we define as MRE multiplicity, the number of times an MRE of the same miRNA family appears on the same transcript. A natural question is how typical is for endogenous target molecules to host MREs with high multiplicity (e.g. with multiplicity between 4 and 7 as in our experimental setup).

To answer to this question we analyzed the database Target Scan Human (Predicted Conserved Targets) [7] and we simply counted on a per-target base the multiplicity of MREs. A summary of the results is displayed in Figure S11 where we show that although a large majority of targets have multiplicity 1 (295.675 MREs out of 369.988), MREs with with larger multiplicity are not so rare (for instance 5500 targets turn out to show a multiplicity between 4 and 7). Note also that typically targets have MREs with different multiplicities for different miRNA families. For instance the MRE with the larger multiplicity in the database is hosted by transcript ONECUT2 and has 20 distinct binding sites for miR-8 and 10 distinct binding sites for miR-320/320abcd.

These results should be taken, as it is often the case with bioinformatic predictions, *cum grano salis* as not all MREs might be simultaneously accessible depending on the biological context (e.g. cell type in which the transcript is expressed) or the state of occupancy of neighboring MREs.

5 Supplemental figures

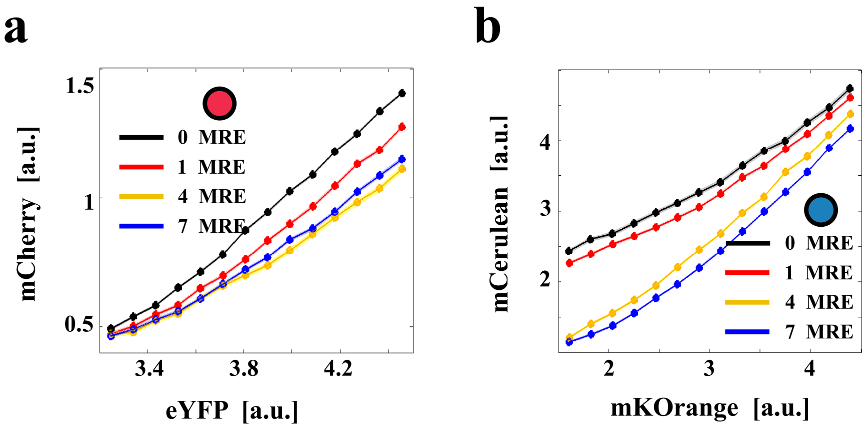

Figure S1: Threshold for single plasmid transfections with $CaCl_2$. (a) mCherry mean fluorescence is plotted against eYFP. A threshold emerges when increasing mCherry MRE. The same holds for mCerulean with respect to mKOrange (b). Shadowed strips around data are the error bars on the biological replicates. For clarity of the image we are not showing the data-dispersion error bars which are similar to those reported in Figure S4. Magenta and cyan circles in legends represent the mCherry and mCerulean fluorophores.

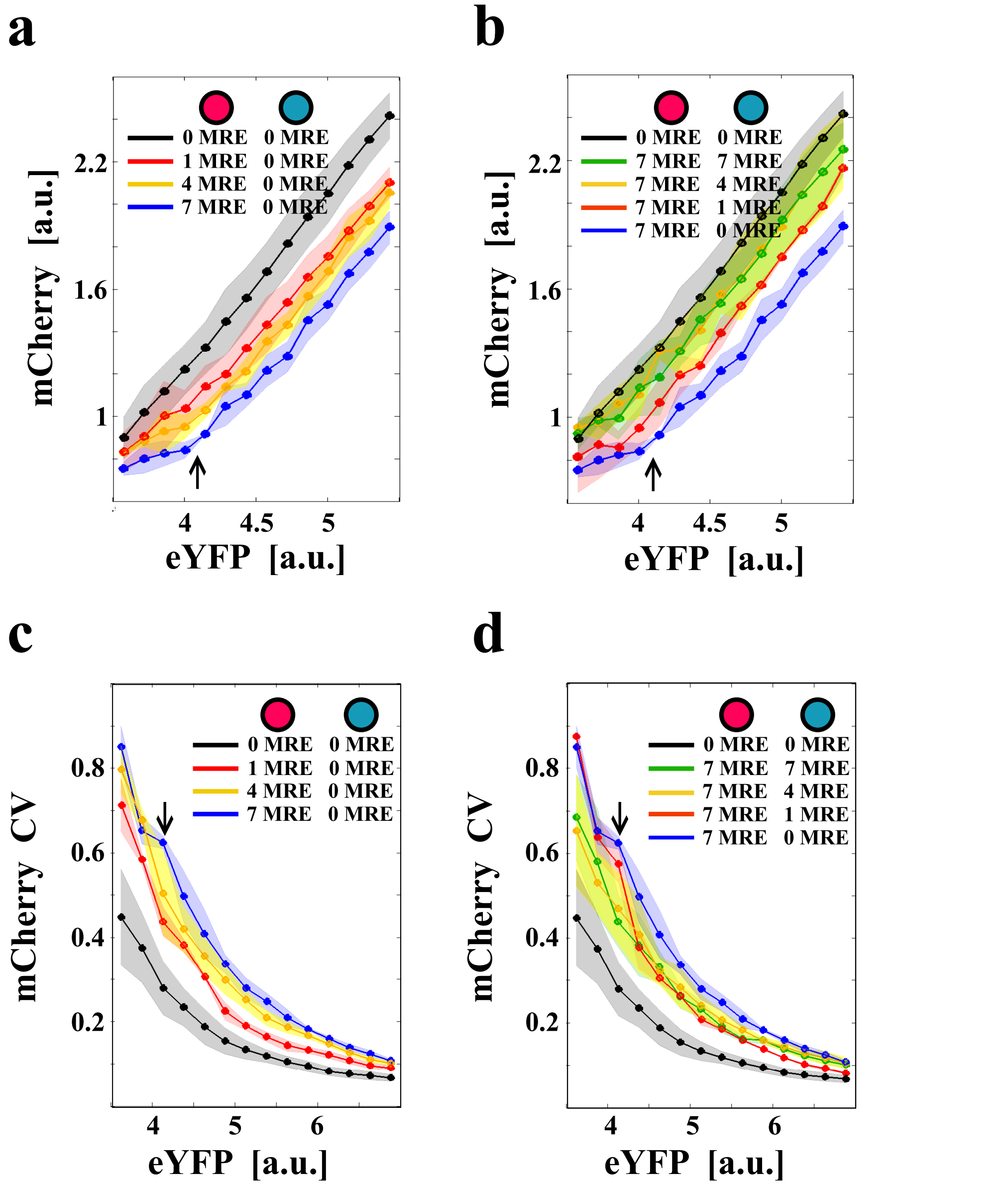

Figure S2: Threshold and noise for $CaCl_2$ transfections. (a-b) mCherry mean fluorescence is plotted against eYFP. Shadowed strips around data are the error bars on the biological replicates. A threshold emerges when increasing mCherry MREs (a) while it disappears when increasing mCerulean MREs (b). (c-d) mCherry coefficient of variation (CV) is plotted against eYFP. Shadowed strips around data are the error bars on the biological replicates. The CV of mCherry globally increases when increasing mCherry MREs (a) while decreases when increasing mCerulean MREs (b). Magenta and cyan circles in legends represent the mCherry and mCerulean fluorophores. Black arrows point to the threshold.

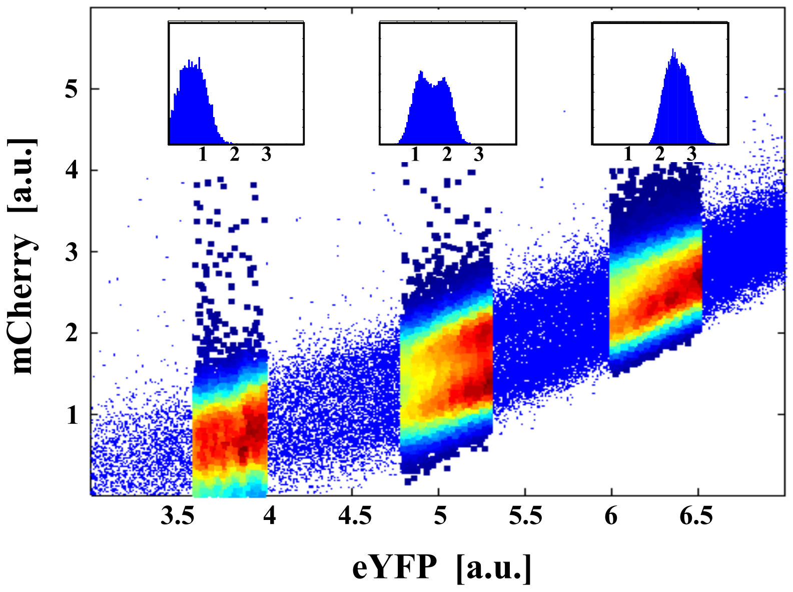

Figure S3: Strong miRNA-target interaction engenders bimodality. Rough mCherry cytofluorimetry data scattered against eYFP. Soon after the threshold two clear phenotypes appear.

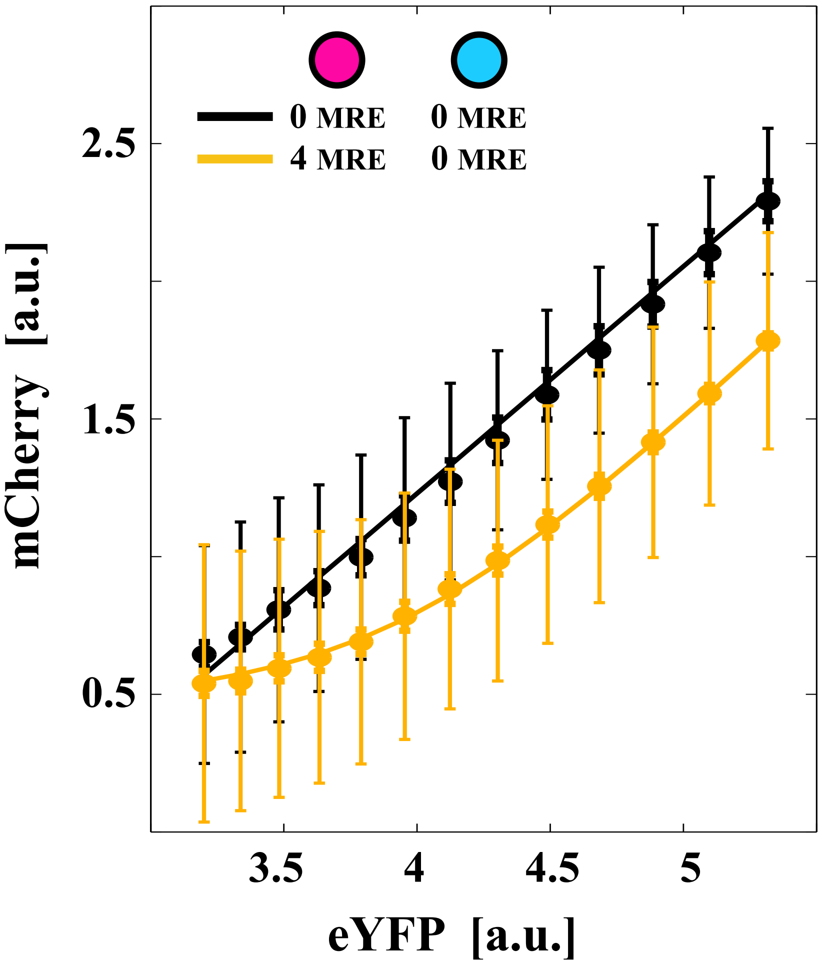

Figure S4: Binned averages for two different constructs. Black and yellow circles are mean values over three experimental replicates for cotransfections with constructs with 0 and 4 miRNA regulatory elements (MRE) on mCherry, respectively (and 0 MRE on mCerulean). Continuous lines are fit of the theoretical model. Magenta and cyan circles represent the two constructs. Data-dispersion (thinner bars) and fluctuation of the mean (thicker bars of almost the symbol size) are superimposed to appreciate the difference between the two error-bar estimates.

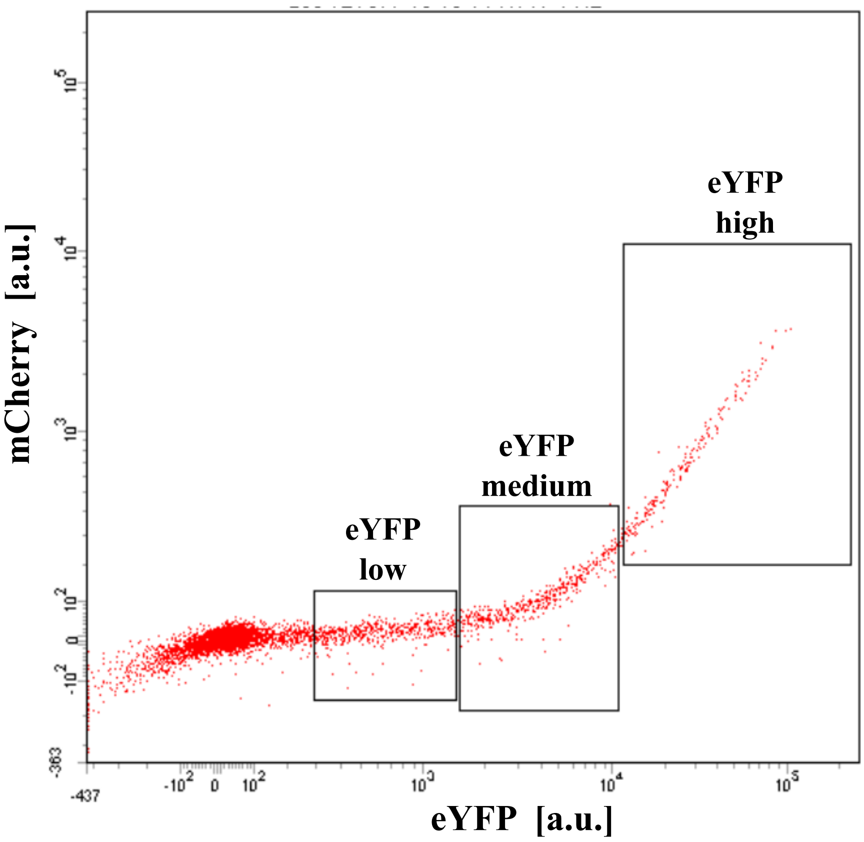

Figure S5: Example of cell sorting for cells co-transfected with pre-miR20a. The scatter plot (eYFP on the x-axis and mCherry on the y-axis) shows how the cells were sorted chosing three intervals based on the eYFP fluorescence level. Each dot is a single cell. The three eYFP intervals (low, medium and high) have been chosen on the basis of the threshold location (below threshold, around threshold and above threshold respectively).

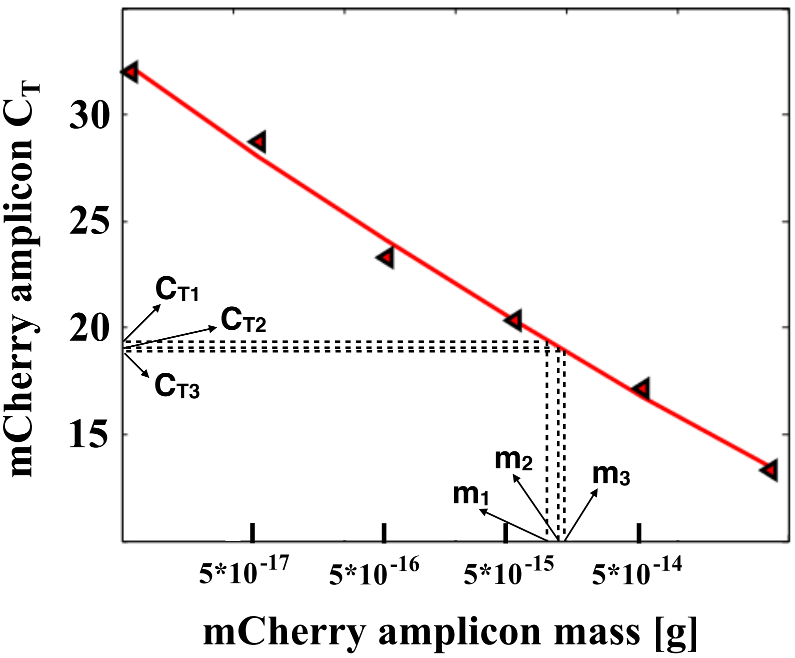

Figure S6: mCherry standard curve obtained from different dilution of the mCherry amplicon. DNA standards were used at amounts ranging from $0.5\,pg$ to $0.005\,fg$ per reaction. The different dilutions of amplicons allowed the definition of a standard curve that directly links the mCherry mass pre-PCR and the threshold cycle (C_T) of the qRT-PCR reactions. C_{T1}, C_{T2} and C_{T3} are the corrected C_Ts for the three technical replicates reported in Table 1 in SI together with their corresponding pre-PCR mass m_1, m_2 and m_3.

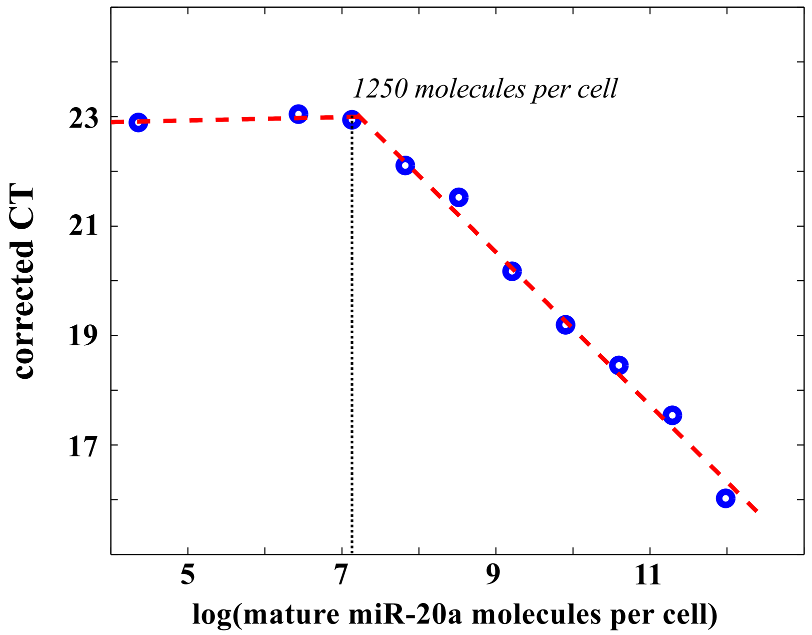

Figure S7: Endogenous miR-20a quantification. Known amounts of mature miR-20a-5p were spiked into cell subpopulations of known numbers in order to obtain a known mean amount of mature miR-20a molecules per cell. The qRT-PCR threshold cycles (C_Ts) of every sample are then directly linked to the mean amount of miR-20a molecules per cell. The changing in slope of the resulting curve corresponds to the amount of endogenous miR-20a molecules per cell (1250 molecules per cell in our case).

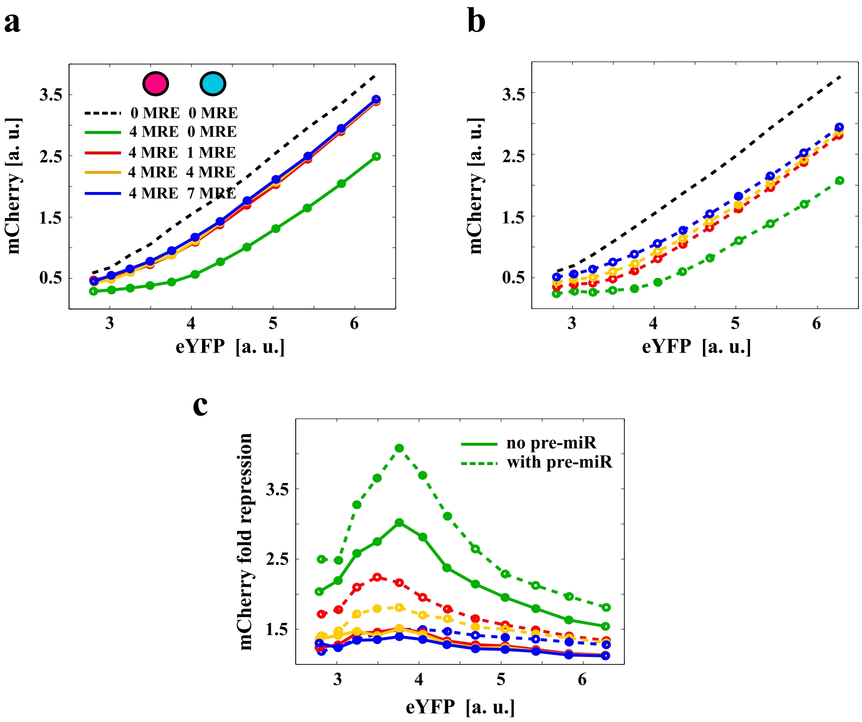

Figure S8: Titration-induced threshold determines the optimal crosstalk. (a-b) mCherry mean fluorescence (a proxy for p_1 in the model) is plotted against eYFP (a proxy for the constitutive expression p_0 in the model) for experiments done in HeLa cells. A threshold (or non-linear behavior) emerges for high number of mCherry MRE, it disappears when increasing mCerulean MRE and can be modulated increasing the pool of available miRNA (panel b corresponds to experiments with 100 nM of pre-miR20a transfected together with the exogenous targets). The intensity of crosstalk (measured in terms of fold-repression F with respect to the unregulated fluorophores) depends on the particular combination of MRE on both exogenous targets and on the miRNA available(c). F is the ratio between the value of mCherry in the absence of miR-20a MRE and its value in the presence of MRE for each eYFP bin and for each N on mCerulean. Purple and cyan circles in legends represent the plasmids coding for the mCherry and mCerulean fluorophores.

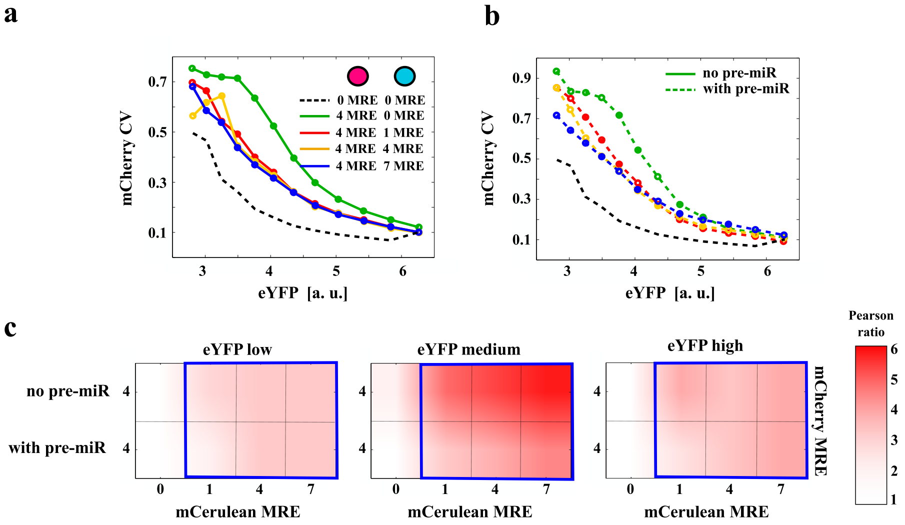

Figure S9: Retroactivity increases cell-to-cell variability and causes target synchronization. (a-b) mCherry total noise quantified by its coefficient of variation CV (a proxy for CV_{p_1} in Figure 1c in the main text) is plotted against eYFP (a proxy for the constitutive expression p_0 in the model) for experiments done in HeLa cells. The CV shows a local maximum for the high miRNA repression (mCherry with 4 MRE and mCerulean with 0 MRE both without (a) and with (b) pre-miR) while decreases when increasing mCerulean MRE number (a-b). Purple and cyan circles in legends represent the plasmids coding for mCherry and mCerulean fluorophores, respectively. In (b) are shown experiments performed with 100 nM of pre-miR-20a transfected together with the exogenous targets. (c) The Pearson ratio is measured for three different values of eYFP basal expression (below, around and above threshold) both in absence and presence of pre-miR. As predicted by the model, the correlation is maximal around the threshold and in this case could be even 6 fold higher than in the unregulated case. Blue-delimited areas are regions whose Pearson ratio (i.e. ratio of Pearson coefficient between mCherry and mCerulean possessing different MRE to the same measure in the absence of MRE) is statistically relevant (p-values < 0.01) with respect to the corresponding unregulated case.

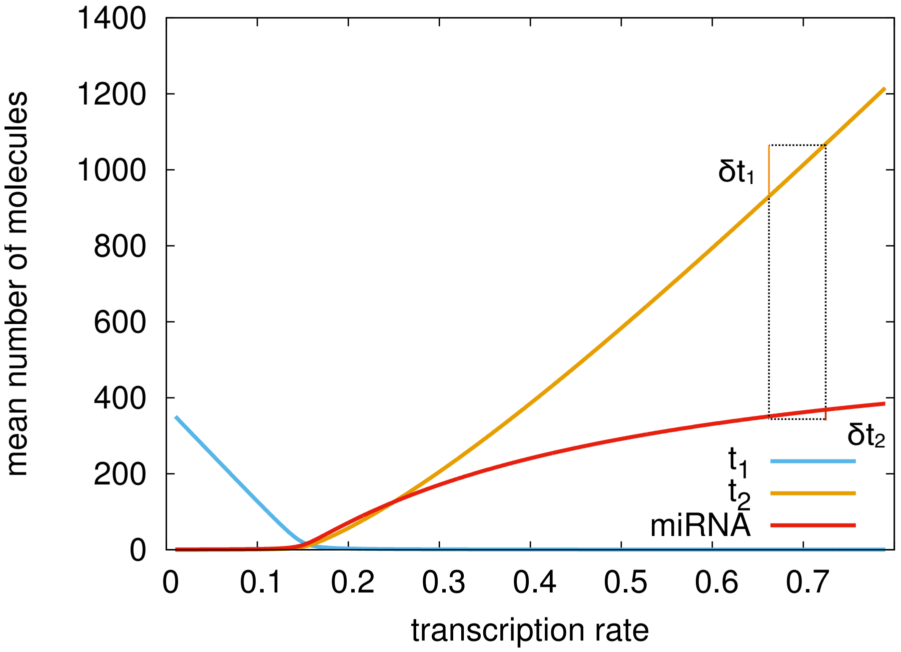

Figure S10: Steady state values for targets and miRNA. Steady state values for the mean number of molecules of targets t_1, t_2 and miRNA s as a function of the target t_1 transcription rate. We display the trend of the system upon varying the transcription rate of t_1 keeping fixed all remaining parameters of the system. In right tail of t_1 and t_2 curves, we show that an increase δ_1 on t_1 will induce a much smaller increase δ_2 on t_2. Curves are obtained by direct integration of Eq.(2).

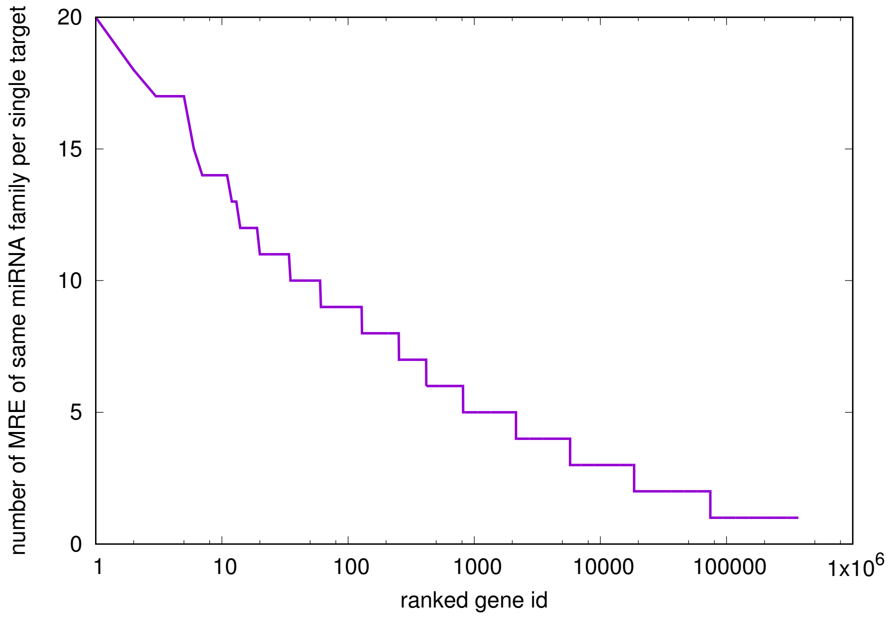

Figure S11: Multiplicity of MREs from Target-Scan. We display the ranked multiplicity of MREs on a per-target base from Target Scan Human (Predicted Conserved Targets). For instance, the leftmost point in the curve indicates that there exists a target hosting up to 20 MREs of a single miRNA family (in this specific case the target gene is ONECUT2 and the miRNA family is miR-9). Note also the logarithmic scale on the x-axys: most of the MREs have multiplicity 1 (295.675 MREs out of 369.988).

References

[1] S Mukherji, MS Ebert, GX Zheng, JS Tsang, PA Sharp, and A van Oudenaarden. MicroRNAs can generate thresholds in target gene expression. *Nat Genet*, 43(9):854–859, 2011.

[2] C Chen, DA Ridzon, AJ Broomer, Z Zhou, DH Lee, JT Nguyen, MB Barbisin, NL Xu, VR Mahuvakar, MR Andersen, KQ Lao, JL Kenneth, and KJ Guegler. Real-time quantification of microRNAs by stem-loop RT-PCR. *Nucleic Acids Research*, 33(20): e179, 2005.

[3] TD Schmittgen, and KJ Livak. Analyzing real-time PCR data by the comparative C_T method. *NATURE PROTOCOLS*, 3(6): 1101–1108, 2008.

[4] N Redshaw, T Wilkes, A Whale, S Cowen, J Huggett, and CA Foy. A comparison of miRNA isolation and RT-qPCR technologies and their effects on quantification accuracy and repeatability. *Biotechniques*, 54(3): 155–64, 2013.

[5] U Ala, FA Karreth, C Bosia, A Pagnani, R Taulli, V Léopold, Y Tay, P Provero, R Zecchina, and PP Pandolfi. Integrated transcriptional and competitive endogenous RNA networks are cross-regulated in permissive molecular environments. *Proc Natl Acad Sci U S A*, 110(18): 7154–7159, 2013.

[6] C Bosia, A Pagnani, and R Zecchina. Modelling competing endogenous RNA networks. *PLoS One*, 8(6): e66609, 2013.

[7] RC Friedman, KKH Farh, CB Burge, and DP Bartel. Most mammalian mRNAs are conserved targets of microRNAs. *Genome Research*, 19(1): 92–105, 2009.
